# Impact of population ageing on cancer-related disability-adjusted life years: A global decomposition analysis

**DOI:** 10.7189/jogh.14.04144

**Published:** 2024-07-19

**Authors:** Juan Zhu, Sainan Li, Xue Li, Le Wang, Lingbin Du, Yanfei Qiu

**Affiliations:** 1 Department of Cancer Prevention, Zhejiang Cancer Hospital, Hangzhou Institute of Medicine, Chinese Academy of Sciences, Hangzhou, Zhejiang, China; 2 Department of Epidemiology and Biostatistics, School of Public Health, Peking University, Beijing, China; 3 School of Public Health and Management, Wenzhou Medical University, Wenzhou, Zhejiang, China

## Abstract

**Background:**

As the global population ages, the burden of cancer is increasing. We aimed to assess the impact of population ageing on cancer-related disability-adjusted life years (DALYs).

**Methods:**

We used the decomposition method to estimate the impact of ageing, population growth, and epidemiological change on cancer-related DALYs from 1990 to 2019, stratified by 204 countries/territories and by their sociodemographic index (SDI). This approach separates the net effect of population ageing from population growth and change in age-specific DALY rates.

**Results:**

Cancer-related DALYs among individuals aged ≥65 years increased by 95.14% between 1990 (52.25 million) and 2019 (101.96 million). Population growth was the main contributor to cancer-related DALYs (92.38 million, attributed proportion: 60.91%), followed by population ageing (41.38 million, 27.28%). Cancer-related DALYs attributed to population ageing followed a bell-shaped pattern when stratified by SDI, meaning they peaked in middle-SDI countries. Cancer-related DALYs attributed to ageing increased in 171 and decreased in 33 countries/territories. The top three cancer types with the highest increase in the absolute number of cancer-related DALYs associated with ageing were tracheal, bronchus, and lung (8.72 million); stomach (5.06 million); and colorectal (4.28 million) cancers, while the attributed proportion of DALYs was the highest in prostate (44.75%), pancreatic (40.93%), and non-melanoma skin (38.03%) cancers.

**Conclusions:**

Population ageing contributed to global cancer-related DALYs, revealing a bell-shaped pattern when stratified by socioeconomic development, affecting middle-SDI countries the most. To respond to the growing ageing population and reduce cancer-related DALYs, it is necessary to allocate health care resources and prioritize interventions for older adults.

The global population is facing significant changes due to population ageing [1,2]. There were an estimated 771 million older adults in 2022, representing 10% of the global population – a 3-fold increase from 258 million in 1980. This trend is expected to continue, with projections indicating that the older population will reach 994 million (12%) by 2030 and 1.6 billion (16%) by 2050 [3]. Further predictions suggest that older adults will comprise 61% of the population in 155 countries by 2100, while by 2050, approximately 1.1 billion older individuals will reside in less developed countries, constituting two-thirds of their population [3,4]. These demographic shifts will strain health care systems worldwide, especially in regions with limited resources, which is why understanding the regional and national implications of population ageing is essential for enhancing health care systems globally. It is also likely to affect the management of chronic diseases such as ischaemic heart disease, stroke, cancer, and diabetes [5-8]. Aside from being a leading cause of mortality, cancer contributes to a substantial proportion of the global disease burden; this proportion is expected to increase in the future, predominantly due to population ageing [9,10]. For example, data from the GLOBOCAN 2020 show that individuals aged >60 years accounted for most cancer diagnoses and deaths globally [11], while cancer registries, including those in China, have reported a marked increase in cancer burden with advancing age, especially among individuals aged 60–79 years [12].

While these challenges could be mitigated through the strategic allocation of health care resources, there is still no comprehensive evaluation of the impact of population ageing on cancer-related disability-adjusted life years. Existing studies have mostly focussed on specific geographical regions [13,14] or selected types of cancer [15−17], limiting their global applicability. This gap hindered international organisations in adapting health care infrastructures to the evolving needs of the ageing population. We thus aimed to assess and quantify the impact of population ageing on cancer-related disability-adjusted life years (DALYs) across 204 countries/territories over three decades (1990–2019), providing strategic insights to guide health care systems in managing cancer amidst global population ageing.

## METHODS

### Data source

DALYs are a comprehensive population health metric that combines years of life lost to premature mortality and years lived with disability due to health loss from disease, injury or other conditions [10]. For our analysis, we retrieved data on cancer-related DALYs from 1990 to 2019 for 204 countries and territories from the Global Burden of Disease (GBD) Study 2019 [18]. These data were collected from systematic reviews, authoritative websites, published reports, and primary data sources such as Demographic and Health Surveys. We defined older adults as individuals aged ≥65 years, following the United Nations classification [3]. Our analysis included 30 cancer causes, classified following the International Classification of Diseases, 10th Revision (Table S1 in the **Online Supplementary Document**). We categorized the study population aged 15–95 into 16 age groups aged.

The sociodemographic index (SDI), which we also retrieved from the GBD database, combines information on the economy, education, and fertility rate of countries around the world, thereby acting as a measure of social and economic development which is closely tied to health outcomes. It is calculated using per capita income, average education achievement, and fertility rates, with the final values of the SDI ranging from 0 to 1. The geometric mean was computed for each location and each year, whereby the 204 countries and territories are classified into five groups based on the SDI quintiles: low, low-middle, middle, high-middle, and high SDI.

### Decomposition analysis

We used a robust decomposition analysis method detailed in previous studies [6–8]. This approach separates the net effect of population ageing from population growth and change in age-specific rates by considering the following three key components: the demographic composition, characterised by a shift toward a larger proportion of older individuals (otherwise known as population ageing); population growth; and age-specific mortality rates. DALY changes can be attributed to population ageing, population growth, and changes in age-specific mortality rates (Text S1 in the **Online Supplementary Document**). The equations for the decomposition analysis were as follows:









































*M_a_, M_p_,* and *M_m_* represent the main effects of population ageing, population growth, and changes in age-specific DALY rates, respectively; *I_pa_, I_pm_, I_am_,* and *I_pam_* denote the two-way and three-way interactions of the three components; and *m_ij_* and *s_ij_* indicate the age-specific DALY rate and proportion of the population for the *i*^th^ age group in the *j*^th^ year (*i* = 1, 2, . . ., 16; *j* = 1, 2), respectively. We calculated these terms using 1990 as the reference year. Additionally, *N*_1_ and *N*_2_ denote population sizes in 1990 and 2019, respectively.

Using decomposition analysis, we quantified the global and national health consequences of population ageing on cancer-related DALYs from 1990 to 2019. Specifically, we calculated the absolute (the number of cancer-related DALYs) and the relative contribution (obtained by dividing attributed cancer-related DALYs by the total DALYs in 1990 and multiplying by 100%). Importantly, this proportion may exceed 100% in cases where the attributed DALYs in a given year exceeded the total DALYs in 1990, indicating an increase in cancer-related DALYs. Conversely, a negative effect denotes a decline in total cancer-related DALYs. Lastly, we used smoothing spline models to examine the relationship between attributed ageing and SDI from 1990 to 2019.

We analysed all data using R, version 4.2.2 (R Core Team, Vienna, Austria). We followed the Guidelines for Accurate and Transparent Health Estimates Reporting (GATHER) statement in presenting our findings (Table S2 in the **Online Supplementary Document**) [19].

## RESULTS

### Population ageing

The number of cancer-related DALYs population among individuals aged ≥65 years increased by 95.14% from 1990 (52.25 million) to 2019 (101.96 million). Stratified by SDI, we observed greater increases in cancer-related DALYs among adults (≥65 years) in the high-, high-middle-, middle-, and low-middle-SDI countries than in the low-SDI countries (high SDI: 19.23 million (48.32%) in 1990 to 29.68 million (58.28%) in 2019; high-middle SDI:15.72 million (34.10%) in 1990 to 28.29 million (44.83%) in 2019; middle SDI: 11.06 million (27.31%) in 1990 to 28.20 million (37.75%) in 2019; low-middle SDI: 4.44 million (24.55%) in 1990 to 11.74 million (30.76%) in 2019; low SDI: 1.78 million (24.94%) in 1990 to 4.00 million (25.97%) in 2019 (**Table 1**)).

**Table 1:** Cancer-related DALYs (in 100 000) in 1990 and 2019, worldwide and by regions, according to the SDI*

			Regions according to SDI									
	**Global**		**High**		**High-middle**		**Middle**		**Low-middle**		**Low**	
	**1990**	**2019**	**1990**	**2019**	**1990**	**2019**	**1990**	**2019**	**1990**	**2019**	**1990**	**2019**
**Total number**	1516.73	2424.12	397.96	509.28	460.92	630.88	404.99	746.92	180.76	381.69	71.38	154.05
**Sex**												
Men	857.34 (56.53)	1361.93 (56.18)	223.11 (56.06)	287.98 (56.55)	274.74 (59.61)	376.04 (59.61)	230.76 (56.98)	434.43 (58.16)	93.70 (51.84)	190.47 (49.90)	34.67 (48.57)	72.33 (46.95)
Women	659.38 (43.47)	1062.19 (43.82)	174.85 (43.94)	221.30 (43.45)	186.18 (40.39)	254.84 (40.39))	174.24 (43.02)	312.49 (41.84)	87.06 (48.16)	191.22 (50.10)	36.71 (51.43)	81.72 (53.05)
**Age in years**												
15–29	88.28 (5.82)	92.91 (3.83)	9.99 (2.51)	6.73 (1.32)	20.91 (4.54)	14.63 (2.32)	33.04 (8.16)	29.88 (4.00)	17.72 (9.80)	26.51 (6.95)	6.57 (9.20)	15.10 (9.80)
30–64	905.94 (59.73)	1311.65 (54.11)	195.69 (49.17)	205.76 (40.40)	282.83 (61.36)	333.40 (52.85)	261.36 (64.53)	435.07 (58.25)	118.66 (65.65)	237.75 (62.29)	47.01 (65.86)	98.94 (64.23)
65–74	332.13 (21.90)	598.93 (24.71)	108.24 (27.20)	147.4 (28.94)	101.76 (22.08)	168.68 (26.74)	77.92 (19.24)	179.29 (24.00)	31.19 (17.25)	76.29 (19.99)	12.87 (18.03)	26.98 (17.51)
75–84	162.31 (10.70)	328.92 (13.57)	68.83 (17.30)	107.72 (21.15)	48.53 (10.53)	89.36 (14.16)	28.65 (7.07)	85.49 (11.45)	11.76 (6.51)	34.97 (9.16)	4.45 (6.23)	11.22 (7.28)
85–95	28.07 (1.85)	91.72 (3.78)	15.21 (3.82)	41.68 (8.18)	6.89 (1.49)	24.81 (3.93)	4.03 (1.00)	17.19 (2.30)	1.44 (0.80)	6.17 (1.62)	0.49 (0.69)	1.82 (1.18)

DALYs – disability-adjusted life years, SDI – socio-demographic index

*Presented as n (%) unless specified otherwise.

### Global cancer-related DALYs attributed to population growth and ageing

**Figure 1:**
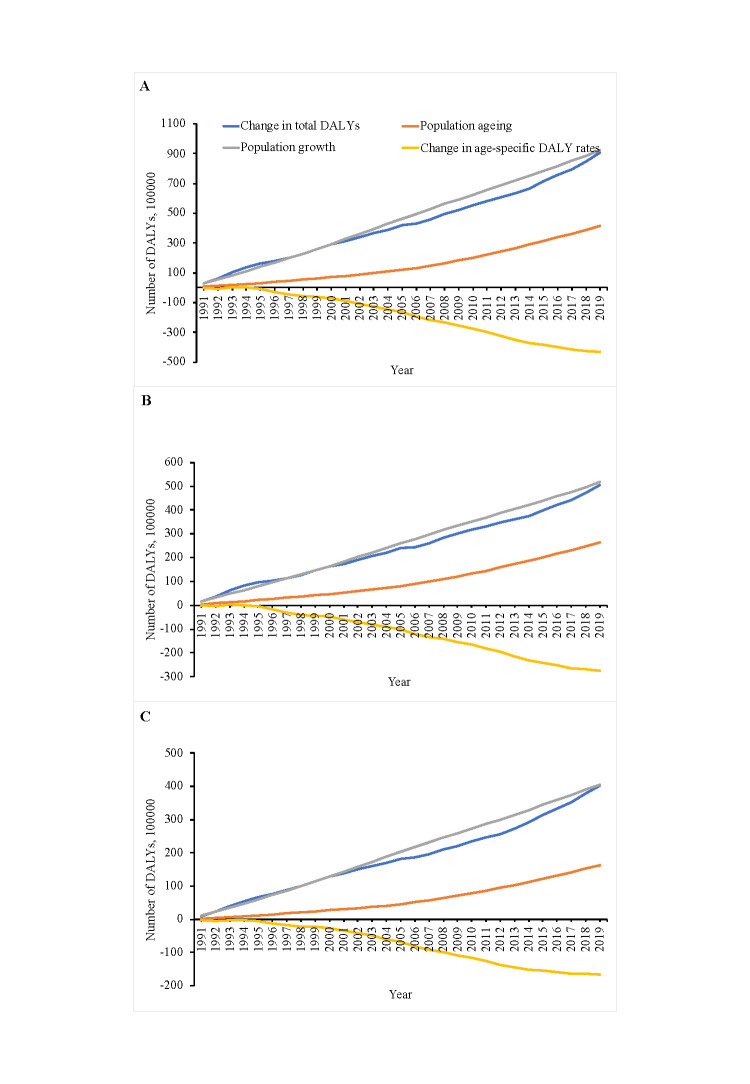
Global changes in cancer-related DALYs attributed to population ageing, population growth and change in age-specific DALY rates from 1991 to 2019. **Panel A.** Global DALYs. **Panel B.** Global DALYs in men. **Panel C.** Global DALYs in women.

Population growth was the main contributor to cancer-related DALYs globally, followed by population ageing. Using the figures from 1990 as a reference for each subsequent year, global cancer-related DALYs attributed to population growth and ageing increased steadily from 1991 to 2019. The numbers of cancer-related DALYs and proportions attributed to population growth, ageing, and epidemical change were 92.38 million (60.91%), 41.38 million (27.28%), and −43.02 million (−28.36%), respectively. The number and attributed proportion of cancer-related DALYs globally due to population growth consistently increased from 1991 to 2019, both in men (2019: 51.69 million (60.29%)) and women (2019: 40.62 million (61.61%)). The corresponding cancer-related DALYs attributed to the global population ageing in 2019 were 26.31 million (30.69%) for men and 16.27 million (24.68%) for women (**Figure 1**, **Table 2**). Our predictions suggest that cancer DALYs attributable to ageing will increase to 74.75 million per 100 000 population by 2030 (Figure S1 in the **Online Supplementary Document**).

**Table 2:** Number of cancer-related DALYs (in 100 000) and the proportion attributed to population ageing by SDI, 1990–2019*

	Population ageing, n (attributed %)	Population growth, n (attributed %)	Change in age-specific DALY rates, n (attributed %)	Change in total DALYs
**Global**				
Men	263.13 (30.69)	516.88 (60.29)	−275.43 (−32.13)	504.58
Women	162.74 (24.68)	406.23 (61.61)	−166.16 (−25.20)	402.81
Total	413.78 (27.28)	923.81 (60.91)	−430.20 (−28.36)	907.39
**High SDI**				
Men	80.38 (36.03)	74.41 (33.35)	−89.92 (−40.30)	64.87
Women	40.13 (22.95)	49.79 (28.47)	−43.47 (−24.86)	46.45
Total	112.12 (28.17)	122.66 (30.82)	−123.46 (−31.02)	111.32
**High-middle SDI**				
Men	96.99 (35.30)	112.55 (40.97)	−108.24 (−39.40)	101.31
Women	50.31 (27.02)	72.43 (38.90)	−54.10 (−29.06)	68.65
Total	140.26 (30.43)	183.88 (39.89)	−154.18 (−33.45)	169.96
**Middle SDI**				
Men	109.04 (47.25)	151.17 (65.51)	−56.54 (−24.50)	203.67
Women	75.92 (43.57)	119.30 (68.47)	−56.96 (−32.69)	138.26
Total	184.51 (45.56)	271.82 (67.12)	−114.41 (−28.25)	341.92
**Low-middle SDI**				
Men	20.19 (21.55)	79.67 (85.02)	−3.09 (−3.30)	96.77
Women	22.10 (25.38)	82.35 (94.58)	−0.29 (−0.34)	104.16
Total	42.88 (23.72)	162.13 (89.69)	−4.08 (−2.26)	200.92
**Low SDI**				
Men	−2.84 (−8.18)	42.35 (122.15)	−1.85 (−5.34)	37.66
Women	−0.63 (−1.72)	47.18 (128.53)	−1.54 (−4.20)	45.01
Total	−3.27 (−4.58)	89.51 (125.40)	−3.58 (−5.01)	82.67

DALYs – disability-adjusted life years, SDI – socio-demographic index

*The attributed proportion was calculated as the change in total DALYs attributed to population ageing between 1990 and 2019 divided by total DALYs in 1990 × 100%. The ratio was calculated as the change in total DALYs attributed to the change in mortality rate divided by that associated with population ageing.

### Cancer-related DALYs attributed to population ageing by SDI

**Figure 2:**
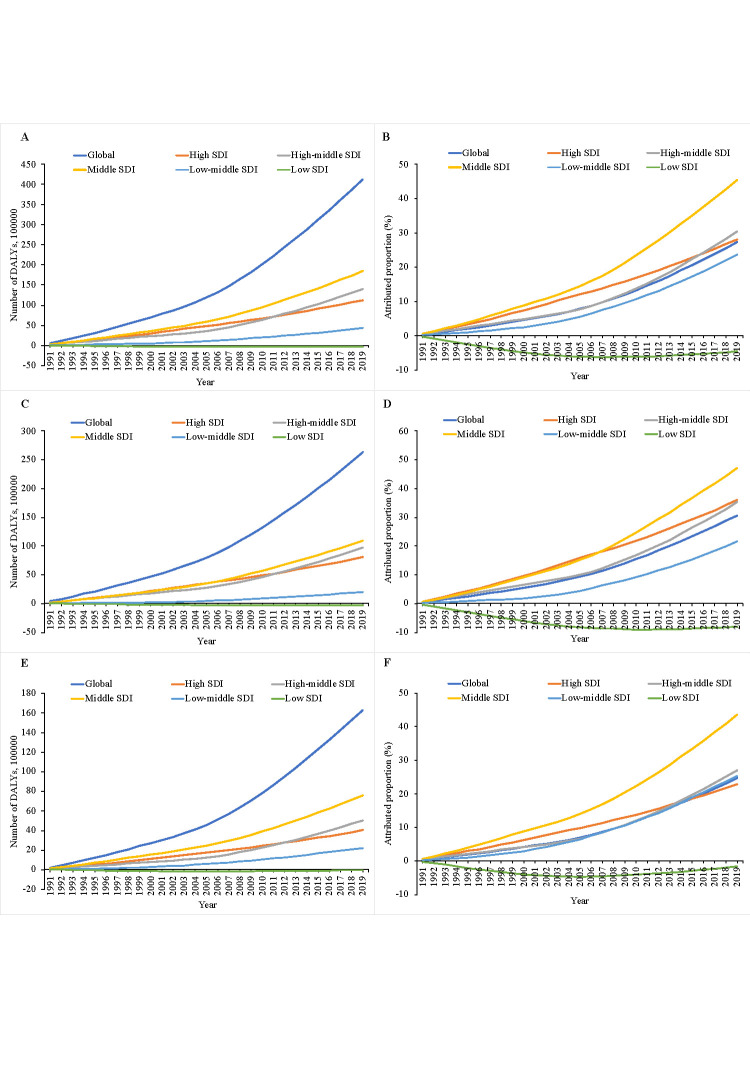
Cancer-related DALYs attributed to population ageing by SDI, 1990–2019. **Panel A.** Number of DALYs attributed ageing. **Panel B.** Proportion of DALYs attributed ageing. **Panel C.** Number of DALYs attributed ageing in men. **Panel D.** Proportion of DALYs attributed ageing in men. **Panel E.** Number of DALYs attributed to ageing in women. **Panel F.** Proportion of DALYs attributed to ageing in women.

Over the past three decades, cancer-related DALYs due to population ageing have steadily increased in high-, upper-middle-, and lower-middle-income countries, with greatest increases in middle-SDI countries and greatest decreases in low-income countries (**Figure 2**, Table S3 in the **Online Supplementary Document**). The cancer-related DALYs attributed to population ageing between 1990 and 2019 were 11.21 million (28.17%), 14.03 million (30.43%), 18.45 million (45.56%), 4.29 million (23.72%), and −0.33 million (−4.58%) for high-, upper-middle-, middle-, lower-middle-, and low-income-SDI countries, respectively (**Table 2**, Figure S2 in the **Online Supplementary Document**). The proportion of cancer-related DALYs due to ageing linked to increased SDI values (*P* < 0.05) showed a bell-shaped curve (Figure S3 in the **Online Supplementary Document**).

We observed similar patterns for men and women (**Figure 2**, Table S4–5 in the **Online Supplementary Document**). Specifically, the cancer-related DALYs attributed to population ageing among men were 8.04 million (36.03%), 9.70 million (35.3%), 10.90 million (47.25%), 2.02 million (21.55%) and −0.28 million (−8.18%) in high-, upper-middle-, middle-, lower-middle-, and low-income countries between 1990 and 2019, respectively. The corresponding cancer-related DALYs (100 000) attributed to ageing among women were 4.01 million (22.95%), 5.03 million (27.02%), 7.59 million (43.57%), 2.21 million (25.38%), and −0.06 million (−1.72%), respectively (**Table 2**).

### Cancer-related DALYs attributed to population ageing in 204 countries/territories

**Figure 3:**
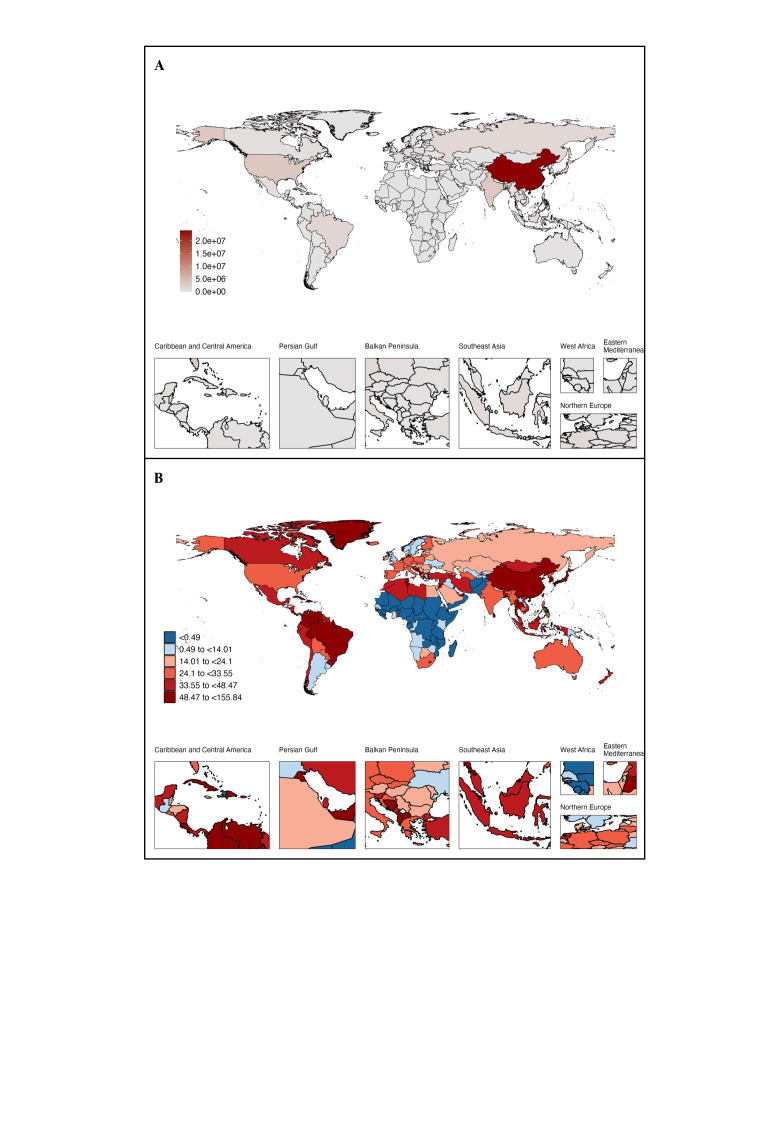
Proportion of DALYs attributed to population ageing between 1990 and 2019 in 204 countries and territories. **Panel A.** Number of DALYs attributed to ageing. **Panel B.** Proportion of DALYs attributed to ageing.

Cancer-related DALYs attributed to ageing increased in 171 countries/territories between 1990 and 2019 (**Table 3**). China saw the highest increase in DALYs due to population ageing (24.00 million). DALYs attributed to ageing worldwide were the highest in the USA (3.27 million), India (3.06 million), Japan (2.87 million), and Brazil (1.56 million). Conversely, cancer-related DALYs attributed to ageing decreased in 33 countries/territories from 1990 to 2019. The highest decrease occurred in Afghanistan, Pakistan, Nigeria, Ethiopia, and Mozambique (**Figure 3**, Table S6 and Figure S4 in the **Online Supplementary Document**). The attributed proportion of DALYs due to ageing varied from −63.36% in Equatorial Guinea to 155.84% in the United Arab Emirates. Population ageing was strongly associated with increased cancer-related DALYs in the United Arab Emirates (155.84%), Northern Mariana Islands (110.22%), Bahrain (93.78%), United States Virgin Islands (78.45%), and the Republic of Korea (77.44%) (**Figure 3**, Table S6 and Figure S4 in the **Online Supplementary Document**).

**Table 3:** Top 10 countries/territories with the highest increase and decrease in cancer-related DALYs attributed to population ageing between 1990 and 2019

	Total		Men		Women	
	**Country/territory**	**Attributed to ageing**	**Country/territory**	**Attributed to ageing**	**Country/territory**	**Attributed to ageing**
**Attributed numbers (in 100 000) population, ordered by rank**						
1	China	240.03	China	157.20	China	84.96
2	USA	32.69	USA	22.36	India	15.84
3	India	30.60	Japan	20.54	USA	12.20
4	Japan	28.74	India	14.44	Japan	10.02
5	Brazil	15.60	Russian Federation	10.74	Brazil	7.20
6	Russian Federation	12.88	Germany	9.21	Indonesia	5.61
7	Republic of Korea	11.06	Brazil	8.27	Thailand	4.38
8	Germany	10.97	Republic of Korea	8.16	Russian Federation	4.31
9	Indonesia	10.32	France	6.59	Republic of Korea	3.61
10	Thailand	10.31	Italy	6.39	Germany	3.48
195	Somalia	−0.09	Burkina Faso	−0.26	Mali	−0.17
196	Chad	−0.13	Guinea	−0.29	Cameroon	−0.17
197	Guinea	−0.13	Uganda	−0.33	Burkina Faso	−0.19
198	Mali	−0.16	Mali	−0.33	Mozambique	−0.25
199	Democratic Republic of the Congo	−0.17	Democratic Republic of the Congo	−0.40	Uganda	−0.33
200	Mozambique	–0.18	Mozambique	−0.42	Democratic Republic of the Congo	−0.35
201	Ethiopia	−0.25	Ethiopia	−0.72	Ethiopia	−0.46
202	Nigeria	−0.33	Pakistan	−1.73	Nigeria	−0.98
203	Pakistan	−0.52	Nigeria	−1.93	Pakistan	−1.27
204	Afghanistan	−0.78	Afghanistan	−1.99	Afghanistan	−1.27
**Attributed proportion, ordered by rank**						
1	United Arab Emirates	155.84	United Arab Emirates	162.25	United Arab Emirates	149.50
2	Northern Mariana Islands	110.22	Northern Mariana Islands	104.85	Northern Mariana Islands	117.57
3	Bahrain	93.78	Bahrain	101.26	Bahrain	85.14
4	United States Virgin Islands	78.45	Republic of Korea	94.25	Taiwan (Province of China)	75.59
5	Republic of Korea	77.44	United States Virgin Islands	93.12	Guam	68.67
6	Djibouti	72.25	Djibouti	90.89	Thailand	67.10
7	Thailand	71.51	Greenland	81.42	Palau	66.46
8	Taiwan (Province of China)	69.45	Singapore	78.39	Kuwait	64.67
9	Singapore	69.30	Venezuela (Bolivarian Republic of)	77.40	Republic of Korea	64.10
10	Guam	68.92	Thailand	75.44	United States Virgin Islands	62.65
195	Burkina Faso	−22.38	Sao Tome and Principe	−30.52	Sao Tome and Principe	−12.36
196	Nigeria	−22.54	Chad	−31.41	Somalia	−15.15
197	Guinea	−23.56	Cabo Verde	−32.18	Mozambique	−18.78
198	Mali	−25.26	Benin	−32.40	Guinea	−20.92
199	Mozambique	−27.31	Sierra Leone	−37.61	Sierra Leone	−22.84
200	Sierra Leone	−29.23	Burkina Faso	−39.05	Mali	−23.59
201	Chad	−33.52	Mozambique	−43.59	Liberia	−28.04
202	Liberia	−43.84	Liberia	−61.80	Equatorial Guinea	−31.18
203	Afghanistan	−61.62	Afghanistan	−88.72	Chad	−35.36
204	Equatorial Guinea	−63.36	Equatorial Guinea	−100.62	Afghanistan	−43.72

For men and women, the top 10 countries/territories with the highest increase and decrease of cancer-related DALYs associated with population ageing were similar. China has the highest number of DALYs due to ageing, and the United Arab Emirates has the highest increase in the proportion of DALYs attributed to population ageing. Whereas the highest decrease of DALYs attributed to population ageing was in Afghanistan and Equatorial Guinea (**Figure 3**, **Table 3**, Table S6 and Figure S4 in the **Online Supplementary Document**).

### The top 10 cancer types with the highest increase in cancer-related DALYs attributed to population ageing

**Figure 4:**
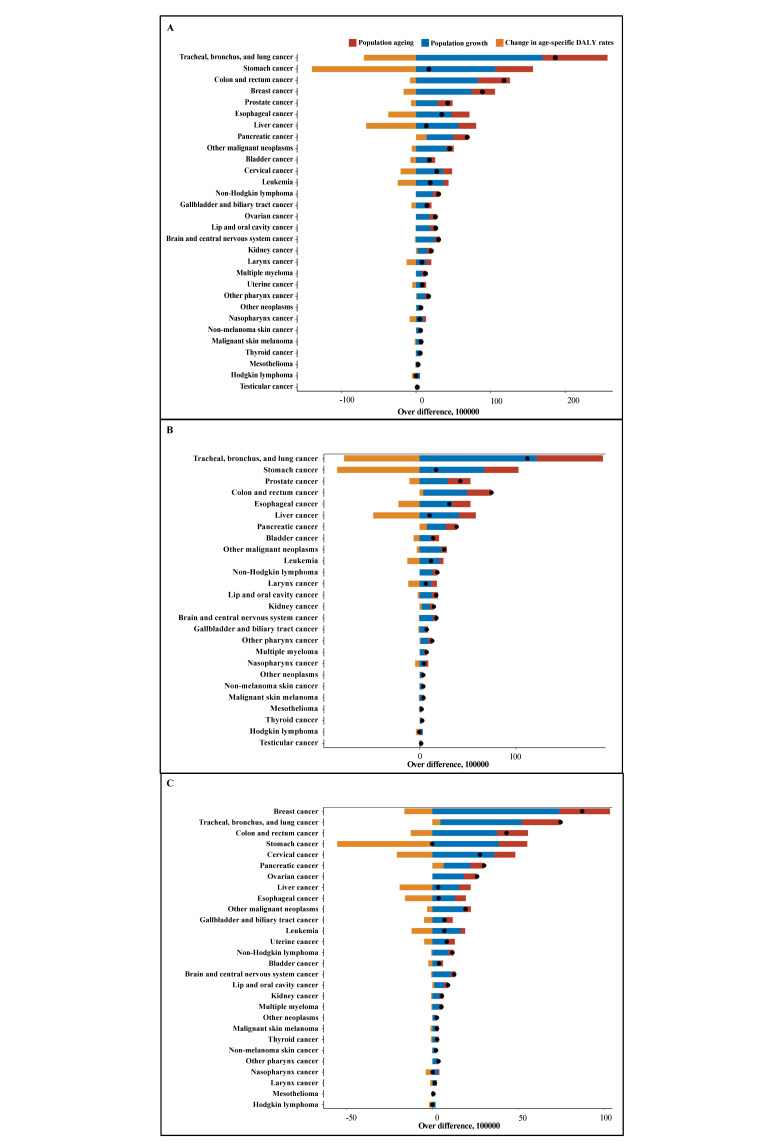
Cancer-related DALYs attributed to population ageing in 30 cancer types, 1990–2019. **Panel A.** Number of DALYs attributed to ageing. **Panel B.** Number of DALYs attributed to ageing in men. **Panel C.** Number of DALYs attributed to ageing in women.

The top 10 cancer types with the highest increase in the absolute number of cancer-related DALYs associated with population ageing between 1990 and 2019 were tracheal, bronchus, and lung (8.72 million); stomach (5.06 million); colorectal (4.28 million); breast (3.13 million); oesophagal (2.42 million); liver (2.38 million); prostate (1.95 million); pancreatic (1.90 million); and cervical cancers (1.25 million) (**Figure 4**, **Table 4**; Table S7 in the **Online Supplementary Document**). Notably, prostate (44.75%); pancreatic (40.93%); non-melanoma skin (38.03%); bladder (37.10%); multiple myeloma (37.03%); kidney (35.32%); colorectal (34.67%); gallbladder and biliary tract (33.97%); and tracheal, bronchus, and lung cancers (32.20%) had the highest increases in the proportion of cancer-related DALYs attributed to ageing. We found similar results for men and women (**Table 4**). Moreover, 13 out of 30 cancer types display the highest DALYs attributed to ageing in middle-SDI regions, such as oesophagal cancer, stomach cancer, and cervical cancer (Figure S5 in the **Online Supplementary Document**), while 16 out of 30 cancer types had the highest DALYs in high-SDI regions, such as tracheal, bronchus, and lung cancer; colorectal cancer; and prostate cancer.

**Table 4:** Top 10 cancer types with the highest increase in cancer-related DALYs attributed to population ageing between 1990 and 2019*

	Total		Men		Women	
	**Cancer causes**	**Attributed to ageing**	**Cancer causes**	**Attributed to ageing**	**Cancer causes**	**Attributed to ageing**
**Attributed numbers (in 100 000), ordered by rank**						
1	Tracheal, bronchus and lung cancer	87.23	Tracheal, bronchus and lung cancer	68.95	Breast cancer	29.53
2	Stomach cancer	50.55	Stomach cancer	35.60	Tracheal, bronchus, and lung cancer	22.51
3	Colon and rectum cancer	42.80	Colon and rectum cancer	25.47	Colon and rectum cancer	18.08
4	Breast cancer	31.30	Prostate cancer	23.37	Stomach cancer	16.50
5	Esophageal cancer	24.23	Esophageal cancer	18.73	Cervical cancer	12.22
6	Liver cancer	23.83	Liver cancer	17.61	Pancreatic cancer	7.93
7	Prostate cancer	19.46	Pancreatic cancer	11.19	Ovarian cancer	7.56
8	Pancreatic cancer	19.00	Bladder cancer	8.18	Liver cancer	6.59
9	Cervical cancer	12.49	Larynx cancer	6.20	Esophageal cancer	6.30
10	Other malignant neoplasms	9.66	Lip and oral cavity cancer	5.98	Other malignant neoplasms	4.18
**Attributed proportion, ordered by rank**						
1	Prostate cancer	44.75	Prostate cancer	53.74	Pancreatic cancer	39.24
2	Other neoplasms	41.97	Other neoplasms	48.68	Other neoplasms	37.40
3	Pancreatic cancer	40.93	Bladder cancer	43.08	Tracheal, bronchus and lung cancer	33.36
4	Non-melanoma skin cancer	38.03	Pancreatic cancer	42.70	Multiple myeloma	33.34
5	Bladder cancer	37.10	Multiple myeloma	41.38	Bladder cancer	32.51
6	Multiple myeloma	37.03	Kidney cancer	39.95	Colon and rectum cancer	30.65
7	Kidney cancer	35.32	Colon and rectum cancer	39.52	Kidney cancer	30.24
8	Colon and rectum cancer	34.67	Gallbladder and biliary tract cancer	39.27	Gallbladder and biliary tract cancer	30.07
9	Gallbladder and biliary tract cancer	33.97	Mesothelioma	37.32	Lip and oral cavity cancer	28.17
10	Tracheal, bronchus and lung cancer	32.20	Breast cancer	36.65	Ovarian cancer	28.00

*The attributed proportion for total was calculated as the number of DALYs attributed to population ageing for each cause of DALY/151.67 million (total DALYs in 1990) × 100%. The attributed proportion for men was calculated as the number of DALYs attributed to population ageing for each cause of DALY/85.73 million (total DALYs for men in 1990) × 100%. The attributed proportion for women was calculated as the number of DALYs attributed to population ageing for each cause of DALY/2.42 million (total DALYs for women in 1990) × 100%.

## DISCUSSION

Our decomposition analysis of the global and national impact of population ageing on cancer-related DALYs from 1990 to 2019 had three key findings. First, the global cancer-related DALYs attributed to population growth and ageing increased rapidly from 1991 to 2019. Second, the increase in cancer-related DALYs due to ageing showed considerable diversity across regions and countries, with a bell-shaped pattern emerging in relation to socioeconomic development, affecting middle-SDI countries the most. Overall, 83.8% of countries were associated with increased DALYs due to ageing, with the highest increase in China (number of DALYs) and the United Arab Emirates (proportion). Third, certain cancer types were disproportionately affected by population ageing. Specifically, tracheal, bronchus, and lung cancer; stomach cancer; and colorectal cancer had the highest increase in DALYs associated with ageing. In contrast, prostate cancer, pancreatic cancer, and non-melanoma skin cancer showed the highest attributed proportions of DALYs due to ageing. These findings highlight the need for countries to adopt tailored preventive and therapeutic strategies for older individuals to effectively manage the rising health care demands resulting from population ageing.

The impact of ageing on DALYs is direct and significant. With an increase in the proportion of elderly individuals in the population, the incidence of age-related chronic diseases (such as cancer, cardiovascular diseases, diabetes, etc.) increases. Elderly individuals, due to declining immune system function and the coexistence of multiple chronic conditions, are more susceptible to health damage, leading to a decline in quality of life and premature death. Therefore, ageing directly increases the loss of healthy life years due to disease and disability, significantly increasing the burden of DALYs on society. The impact of population ageing can be attributed to population growth, increases in life expectancy, and decreases in fertility [3,20]. First, the global population growth rate, which was 2.1% annually in 1962–65, has declined to <1% in 2020 and is expected to decrease further to approximately 0.5% by 2050. Countries like Japan and South Korea are experiencing population declines, and China reported its first negative population growth in 2022 [21–23]. Second, following consistent increases in recent years, the global average life expectancy is projected to rise from 72.8 years in 2019 to 77.2 years by 2050. Lastly, the global average fertility rate has decreased from 5.0 in 1950 to 2.3 in 2021, with projections suggesting further reductions in the future. These trends collectively contribute to the exacerbation of population ageing [3,20], emphasising the importance of initiatives promoting healthy ageing to prevent and control cancer.

According to the World Health Organization report, East and Southeast Asia, Latin America, and the Caribbean are experiencing the fastest rates of population ageing [3]. These regions, including countries such as China, Korea, Japan, Thailand, and Singapore, also saw the highest increases in cancer burden in our study. Interestingly, population ageing was linked to a decrease in cancer-related DALYs in several low-SDI countries such as Afghanistan, Guinea, and Liberia. This decrease may be influenced by data reporting quality; diagnostic and treatment capacities; sociopolitical stability; and health care conditions, rather than a true decline in cancer burden. We further observed a bell-curved relationship, with a transitional stage where middle SDI countries are most affected. First, the disease burden in middle-income countries is mostly transitioning from a high burden of infectious diseases to a high burden of chronic diseases. Specifically, infectious diseases become more effectively controlled with socioeconomic developments, while lifestyle changes (such as urbanisation and Westernised diets) significantly increase cancer risk. Therefore, non-communicable diseases (such as cancer) have become major health threats. These diseases are more common in the elderly population, thereby exacerbating the health burden associated with ageing [24]. Second, while middle-SDI countries are experiencing rapid economic development, their social welfare systems, health care resource allocation, and infrastructure construction may not fully keep pace with population ageing. This leads to issues with health care accessibility, health insurance coverage, and pensions, with coverage still incomplete and protection levels limited [25]. The most populated middle-SDI countries, such as India, have a large and rapidly ageing population, facing significant income disparity, uneven regional development and limited resources in the public system. Further expanding health insurance coverage, particularly in rural and impoverished areas, could ensure more people can afford cancer treatment. Healthcare systems need to prepare for and manage the substantial impact of population ageing, especially in highly populated middle-SDI countries with an existing cancer epidemic.

A study focussing on fractions and trends of cancer deaths attributable to population ageing in China used a different methodological approach, whereby the authors decomposed four components: population ageing, population growth, age-specific incidence, and deaths due to case fatality rates [14]. The core indicator was cancer deaths due to ageing, and their findings are consistent with our study. Another decomposition analysis of global cancer occurrence used the decomposition method as we did in our study, but with the analysis indicators being incidence and mortality; they found that the global increase in cancer cases and deaths was mainly attributed to population ageing [6]. The greatest attribution in our study, in turn, was to population growth. Additionally, other studies using decomposition analysis of DALYs and deaths associated with liver cancer [26], diabetes [7], ischemic stroke [27], and the top 10 causes of global death [5] had consistent results, with the largest attribution coming from population growth, followed by population ageing. This highlights how ageing in upper to middle-income countries and population growth in low to middle-income countries hinder efforts to reduce the burden of disease.

Based on the analysis of cancer types, we observed that lung and gastrointestinal cancers are related to the highest increase in the number of cancer-related DALYs associated with ageing, mainly resulting from high incidence and mortality globally. Some effective prevention measures include strengthening smoking cessation programmes for the elderly to reduce the impact of tobacco exposure on lung cancer incidence and improving the living environment of the elderly to reduce exposure to air pollution and harmful substances. Regular lung cancer check-ups for high-risk elderly individuals for early detection and intervention are also recommended. Stomach cancer, meanwhile, has a high incidence in East Asian countries, such as China, Japan, and Korea. With global population ageing, the incidence and mortality rates of gastric cancer in the elderly are significantly increasing, leading to a higher DALY burden. Prevention measures could include the eradication treatment of *Helicobacter pylori*; reducing the intake of high-salt or pickled foods; and increasing the consumption of fruits and vegetables. Considering the invasiveness and low compliance of endoscopy, it might also be necessary to explore novel screening technologies such as magnetic capsule endoscopy, or to develop innovative and feasible non-invasiveness biomarkers for gastric cancer diagnosis, such as cell-free DNA, DNA methylation, or exosomes. Notably, prostate and pancreas cancer contributed the highest attributed proportion due to ageing, aligning with findings of studies on the occurrence and mortality of cancer in ageing populations [6]. Therefore, tailored preventative and therapeutic strategies focussing on the specific cancer types most affected by population ageing are essential.

The impact of ageing on cancer-related DALYs differs between men and women, largely due to the incidence and disease burden of gender-specific cancers. For example, breast cancer is specific to women and has a high incidence rate among elderly women; prostate cancer is specific to men, and its incidence increases significantly in elderly men. Compared with elderly women, elderly men may face less family care and social support, leading to lower adherence to disease management, and they may also be more significantly impacted by occupational exposures (e.g. smoking, occupational carcinogens). Establishing social support networks for this population by providing psychological counselling and family care support could help address these issues. Moreover, men may be less likely to seek preventive health care services due to cultural or personal factors, particularly in old age, resulting in late cancer diagnoses and poorer treatment outcomes. While younger women may frequently utilise prenatal and gynaecological check-ups, elderly women, particularly in low-income countries, may have reduced access to such services [28], as these systems are more likely to face health care resource shortages. Screening programmes for breast and cervical cancers should be maintained or redesigned for sustainability, ensuring elderly women have access to necessary screening and preventive services.

Cultural attitudes towards ageing and cancer treatment in different regions likewise affect DALYs. For example, they may influence early detection and willingness to seek treatment [29], whereby people may lack sufficient awareness of health check-ups and cancer screening, leading to late cancer detection. Increasing health education and promotion can change this attitude and increase participation in early screening programmes. In some Asian countries, cultural taboos surrounding illness may discourage people from undergoing routine checks. Cultural distrust of modern medicine, cultural beliefs, traditional customs, religious views, folk remedies, and preferences for traditional treatment methods may delay early cancer diagnosis and intervention with modern treatments. For example, rapid ageing in Japan and the emphasis on health in traditional culture, along with the universal health care system, resulted in higher participation rates in early screening and treatment, as was the case with the national gastric cancer screening programme [30]. Conversely, in India, cultural diversity; the coexistence of traditional and modern medicine; taboos and sensitivities surrounding illness in traditional customs; religious views; and distrust of modern medicine may lead to delayed medical visits [31].

An in-depth examination of health care system variations is essential for addressing the challenges posed by the burden of serious diseases. China’s successful policy and health care reform, which has expanded the coverage and reimbursement rates of medical insurance, especially for the high costs of cancer treatment, has provided more support to citizens and reduced poverty due to illness. This has improved accessibility to medical services, particularly for rural and low-income populations, such as through the New Rural Cooperative Medical Scheme. Densely populated regions have struggled to establish private health insurance policies [3,4,32], with the growing elderly population imposing budgetary challenges for families and societies, as have costs related to diagnostics and treatment, drugs, hospitalisation and care. These challenges have been exacerbated by a related loss of productivity, growing burdens on caregivers, and social welfare costs. Future economic research should use novel health economic evaluation methods to explore the cost-effectiveness of various strategies for cancer prevention, early screening, treatment, and rehabilitation in middle-SDI countries. These could include evaluations of the applicability and feasibility of these strategies to optimise resource allocation and enhance the sustainability of health care systems, as well as cost-effectiveness or cost-utility analyses to quantify the impact of this burden on the national economy and health care systems, ensuring long-term economic and social benefits.

There are several possible targeted health care strategies for tackling these challenges [33]. The first option is prevention strategies, governments could develop or strengthen laws and regulations, such as public smoking bans; implement environmental governance measures; promote the widespread vaccination against human papillomavirus (HPV) and hepatitis B to prevent related cancers such as cervical and liver cancer; or enhance vaccination coverage through free or low-cost immunisation programs. Another approach is the establishment of screening programmes, where governmental bodies could implement national cancer screenings and early detection programs for older adults (<75 years); formulate clear screening guidelines and ensure the accessibility of screening programs; or launch mobile screening services in rural and remote areas to ensure early detection opportunities for these populations. They could also collaborate with non-governmental organisations to provide technical and financial support. Lastly, they should further develop cancer screening plans tailored to local conditions. For resource-limited, but high-incidence regions, health care authorities could focus on low-cost cancer screening options and building basic medical service capabilities. The adoption of telemedicine and digital health solutions can likewise enhance care access, particularly for older patients with mobility issues. A third possible avenue could be health care infrastructure enhancements, whereby health care stakeholders could establish or upgrade cancer treatment centres in the most burdened areas, equipped with advanced diagnostic and treatment facilities, or increase training for oncologists, nurses, and other relevant health care professionals to enhance their diagnostic and treatment capabilities. They could also act by improving diagnostic accuracy and accelerating the diagnostic process by introducing the application of artificial intelligence and ensuring equitable distribution of medical resources, especially in rural and remote areas, to reduce disparities in medical services. Lastly, promoting collaborations among government agencies, health care providers, and advocacy organisations is crucial to effectively addressing the multifaceted challenges of an ageing population.

Cancer screening offers various benefits in reducing cancer risk and premature death for the entire population [34–36]. However, in older adults, concerns arise that the potential disadvantages of screening might outweigh these benefits. Screening detects slow-growing cancers that may require ten or more years to appear. Older individuals, therefore, face a higher competing risk of cancer-unrelated death, diminishing their potential advantages, and they may also experience psychological stress due to positive screening results and unnecessary diagnostic interventions. Moreover, over-screening is common with such screening programmes, occurring when the life expectancy in older adults is less than 10–15 years or when they exceed the recommended age thresholds of screening [37,38]. For example, a nationwide study in the USA found that over half of older individuals had recently undergone unnecessary screenings for breast, colorectal, and cervical cancer [39], indicating a trend towards over-screening in this age group. The specific benefits and harms of screening in older adults are often underestimated due to the extrapolation of indirect evidence from younger populations, as most clinical trials exclude participants aged >75 years and as screening guidelines offer limited evidence-based recommendations for discontinuation [40]. More inclusive screenings and real-world research involving older adults could help address these gaps.

The main strength of this study is our quantification of the influence of ageing on global and national cancer-related DALYs between 1990 and 2019. This rigorous decomposition analysis enabled us to make cross-country comparisons and identify countries at high risk of cancer burden due to population ageing. Moreover, we included 30 cancer types in our analysis, and also explored the relationship between the attributed ageing proportion and the SDI, providing valuable insights into health care policies promoting healthy ageing. However, this study also has some limitations. First, population ageing results from declining fertility and increasing life expectancy [41], yet our analysis did not differentiate between these factors. Second, data gaps in the GBD data set, primarily due to limited high-quality country-level data, especially in resource-poor, low-income countries, constrained our study. Future research could address these gaps by using more rigorous, higher-quality data collection approaches.

## CONCLUSIONS

Population ageing contributed to global cancer-related DALYs significantly between 1990 and 2019, with cancer-related DALYs attributed to ageing continuing to rise. The increase due to ageing varied considerably across regions and countries, showing a bell-shaped pattern when analysed by socioeconomic development, whereby middle-SDI countries are affected the most. We observed increased DALYs due to ageing in 83.8% of countries, with the highest increases in China (numbers) and the United Arab Emirates (proportion). Globally, specific cancer types, particularly tracheal, bronchus, and lung cancer, stomach cancer, and colorectal cancer were disproportionately affected by population ageing, with the highest attributed proportions of DALYs due to ageing seen in prostate, pancreatic, and non-melanoma skin cancers. These findings provide a valuable foundation for tailored health care strategies and resource allocation to address this critical public health issue.

## Additional material


Online Supplementary Document
