# Supplementary material for: Impact of population ageing on cancer-related disability-adjusted life years: A global decomposition analysis
Source: J Glob Health. 2024 Jul 19;14:04144. doi: 10.7189/jogh.14.04144 (PMC11259023; doi:10.7189/jogh.14.04144)

Online Supplementary Document

Table S1 List of International Classification of Diseases (ICD-10) codes mapped to the Global Burden of Disease cause list for neoplasms. 2

Text S1 The decomposition method 4

Table S2 Checklist of information that should be included in new reports of global health estimates 8

Table S3 The number (100000) and proportion of cancer-related DALYs attributed to population aging globally and by SDI from 1991 to 2019. 10

Table S4 The number (100000) and proportion of cancer-related DALYs attributed to population aging in males globally and by SDI from 1991 to 2019. 12

Table S5 The number (100000) and proportion of cancer-related DALYs attributed to population aging in females globally and by SDI from 1991 to 2019. 14

Table S6 The number (100000) and proportion of cancer-related DALYs attributed to population aging in 204 countries and territories, 1990 and 2019. 16

Table S7 The number (100000) and proportion of cancer-related DALYs attributed to population aging in 30 cancer types, 1990-2019. 24

Figure S1 The forecast of the number (100000) cancer-related DALYs attributed to population aging globally by 2030. 26

Figure S2 Cancer-related DALYs attributed to population aging, population growth, and change in age-specific DALY rates globally and by SDI, 1990-2019. (a) DALYs; (b) DALYs in males; (c) DALYs in females. 27

Figure S3 The association between cancer-related DALYs attributed to population aging in 204 countries and territories and SDI. (a) Total; (b) Males; (c) Females. 28

Figure S4 DALYs attributed to population ageing between 1990 and 2019 in 204 countries and territories. (a) Number of DALYs attributed to aging in males; (b) Proportion of DALYs attributed to aging in males; (c) Number of DALYs attributed to aging in females; (d) Proportion of DALYs attributed to aging in females. 29

Figure S5 Cancer-related DALYs attributed to population aging by SDI 1990-2019, stratified by 30 cancer types. 30

Table S1 List of International Classification of Diseases (ICD-10) codes mapped to the Global Burden of Disease cause list for neoplasms.

Cause	ICD10
Neoplasms	C00-C13.9, C15-C22.8, C23-C25.9, C30-C34.9, C37-C38.8, C40-C41.9, C43-C45.9, C47-C54.9, C56-C57.8, C60-C63.8, C64-C67.9, C68.0-C68.8, C69.0-C69.8, C70-C73.9, C75-C75.8, C81-C86.6, C88-C91.0, C91.2-C91.3, C91.6, C92-C92.6, C93-C93.1, C93.3, C93.8, C94-C96.9, D00.1-D00.2, D01.0-D01.3, D02.0-D02.3, D03-D06.9, D07.0-D07.2, D07.4-D07.5, D09.0, D09.2-D09.3, D09.8, D10.0-D10.7, D11-D12.9, D13.0-D13.7, D14.0-D14.3, D15-D16.9, D22-D24.9, D26.0-D27.9, D28.0-D28.1, D28.7, D29.0-D29.8, D30.0-D30.8, D31-D36, D36.1-D36.7, D37.1-D37.5, D38.0-D38.5, D39.1-D39.2, D39.8, D40.0-D40.8, D41.0-D41.8, D42-D43.9, D44.0-D44.8, D45-D47.9, D48.0-D48.6, D49.2-D49.4, D49.6, K62.0-K62.1, K63.5, N60-N60.9, N84.0-N84.1, N87-N87.9
Lip and oral cavity cancer	C00-C08.9, D10.0-D10.5, D11-D11.9
Nasopharynx cancer	C11-C11.9, D10.6
Other pharynx cancer	C09-C10.9, C12-C13.9, D10.7
Oesophageal cancer	C15-C15.9, D00.1, D13.0
Stomach cancer	C16-C16.9, D00.2, D13.1, D37.1
Colon and rectum cancer	C18-C21.9, D01.0-D01.3, D12-D12.9, D37.3-D37.5
Liver cancer	C22-C22.8, D13.4
Gallbladder and biliary tract cancer	C23-C24.9, D13.5
Pancreatic cancer	C25-C25.9, D13.6-D13.7
Larynx cancer	C32-C32.9, D02.0, D14.1, D38.0
Tracheal, bronchus, and lung cancer	C33-C34.9, D02.1-D02.3, D14.2-D14.3, D38.1
Malignant skin melanoma	C43-C43.9, D03-D03.9, D22-D23.9, D48.5
Non-melanoma skin cancer	C44-C44.9, D04-D04.9, D49.2
Breast cancer	C50-C50.9, D05-D05.9, D24-D24.9, D48.6, D49.3
Cervical cancer	C53-C53.9, D06-D06.9, D26.0
Uterine cancer	C54-C54.9, D07.0-D07.2, D26.1-D26.9
Ovarian cancer	C56-C56.9, D27-D27.9, D39.1
Prostate cancer	C61-C61.9, D07.5, D29.1, D40.0
Testicular cancer	C62-C62.9, D29.2-D29.8, D40.1-D40.8
Kidney cancer	C64-C65.9, D30.0-D30.1, D41.0-D41.1

Bladder cancer	C67-C67.9, D09.0, D30.3, D41.4-D41.8, D49.4
Brain and central nervous system cancer	C70-C72.9
Thyroid cancer	C73-C73.9, D09.3, D09.8, D34-D34.9, D44.0
Mesothelioma	C45-C45.9
Hodgkin lymphoma	C81-C81.9
Non-Hodgkin lymphoma	C82-C86.6, C96-C96.9
Multiple myeloma	C88-C90.9
Leukaemia	C91-C91.0, C91.2-C91.3, C91.6, C92-C92.6, C93-C93.1, C93.3, C93.8, C94-C95.9
Other malignant neoplasms (internal)	C17-C17.9, C30-C31.9, C37-C38.8, C48-C48.9, C4A, C51-C52.9, C57-C57.8, C60-C60.9, C63-C63.8, C66-C66.9, C68.0-C68.8, C75-C75.8, D07.4, D09.2, D13.2-D13.3, D14.0, D15-D16.9, D28.0-D28.1, D28.7, D29.0, D30.2, D30.4-D30.8, D31-D31.9, D35-D35.2, D35.5-D36, D36.1-D36.7, D37.2, D38.2-D38.5, D39.2, D39.8, D41.2-D41.3, D44.1-D44.8, D48.0-D48.4
Other neoplasms	D32-D33.9, D35.3-D35.4, D42-D43.9, D45-D47.9, D49.6, K62.0-K62.1, K63.5, N60-N60.9, N84.0-N84.1, N87-N87.9

Text S1 The decomposition method

All decomposition methods for absolute numbers attribute differences or changes in total DALYs to the changes in various components or factors, such as population size, age structure, and changes in DALY rates. Compared to the two most commonly used decomposition methods, the new decomposition method we adopted in this study was reported to be robust to the choice of decomposition order of the three factors and the choice of the reference group [1].

Using the difference in total DALYs in 1990 and 2019 for the world, we demonstrate the calculation of DALYs attributed to the three factors. Age was divided using 5-year increments, from 15 years old to 94 years. Let d_{ij}, n_{ij}, m_{ij}, and s_{ij} denote the number of DALYs, population size, age-specific DALY rates, and proportion of population for the ith age group of the jth year, respectively, (i = 1, 2, …, 16; j = 1, 2). Let D_1 and D_2, N_1 and N_2, M_1 and M_2 represent the total number of DALYs, population size, and crude DALY rate for years 1990 and 2019, respectively.

Meaning of mathematical symbols in the decomposition formula

Age group	1990 (j=1)				2019 (j=2)			
	DALY	Population	DALY rates	Age structure	DALY	Population	DALY rates	Age structure
15-19	d_{11}	n_{11}	m_{11}	s_{11}	d_{12}	n_{12}	m_{12}	s_{12}
20-24	d_{21}	n_{21}	m_{21}	s_{21}	d_{22}	n_{22}	m_{22}	s_{22}
25-29	d_{31}	n_{31}	m_{31}	s_{31}	d_{32}	n_{32}	m_{32}	s_{32}
⋮	⋮	⋮	⋮	⋮	⋮	⋮	⋮	⋮
90-94	d_{161}	n_{161}	m_{161}	s_{191}	d_{162}	n_{162}	m_{162}	s_{162}
Total	D_1	N_1	M_1	S_1=1	D_2	N_2	M_2	S_2=1

$D_1 = \sum_{i=1}^{16} d_{i1}$

$D_2 = \sum_{i=1}^{16} d_{i2}$

$N_1 = \sum_{i=1}^{16} n_{i1}$

$N_2 = \sum_{i=1}^{16} n_{i2}$

$M_1 = D_1/N_1$

$M_2 = D_2/N_2$

$m_{ij} = d_{ij}/n_{ij}$

$s_{ij} = n_{ij}/N_j$

Using M_p, M_a, and M_m to represent the main effects of the changes in population size, age structure, and DALY rates, and I_{pa}, I_{pm}, I_{am}, and I_{pam} to represent their two-way and three-way interactions, respectively. These terms are calculated as follows when using the year 1990 as the reference:

$$M_p = \sum_{i=1}^{16}(N_2 - N_1)s_{i1}m_{i1}$$

$$M_a = \sum_{i=1}^{16} N_1(s_{i2} - s_{i1})m_{i1}$$

$$M_m = \sum_{i=1}^{16} N_1 s_{i1}(m_{i2} - m_{i1})$$

$$I_{pa} = \sum_{i=1}^{16}(N_2 - N_1)(s_{i2} - s_{i1})m_{i1}$$

$$I_{pm} = \sum_{i=1}^{16}(N_2 - N_1)s_{i1}(m_{i2} - m_{i1})$$

$$I_{am} = \sum_{i=1}^{16} N_1(s_{i2} - s_{i1})(m_{i2} - m_{i1})$$

$$I_{pam} = \sum_{i=1}^{16}(N_2 - N_1)(s_{i2} - s_{i1})(m_{i2} - m_{i1})$$

Using the year 2019 as the reference, the formulas are calculated as follows:

$$M'_p = \sum_{i=1}^{p}(N_1 - N_2)s_{i2}m_{i2}$$

$$M'_a = \sum_{i=1}^{p} N_2(s_{i1} - s_{i2})m_{i2}$$

$$M'_m = \sum_{i=1}^{p} N_2 s_{i2}(m_{i1} - m_{i2})$$

$$I'_{pa} = \sum_{i=1}^{p}(N_1 - N_2)(s_{i1} - s_{i2})m_{i2}$$

$$I'_{pm} = \sum_{i=1}^{p}(N_1 - N_2)s_{i2}(m_{i1} - m_{i2})$$

$$I'_{am} = \sum_{i=1}^{p} N_2(s_{i1} - s_{i2})(m_{i1} - m_{i2})$$

$$I'_{pam} = \sum_{i=1}^{p}(N_1 - N_2)(s_{i1} - s_{i2})(m_{i1} - m_{i2})$$

The contribution of each factor includes its main effect and partial interactions with other factors.

(1) Suppose *a*%, *b*%, and *c*% of the two-way interaction between population size and age structure, population size and DALY change, and age structure and DALY change are allocated to the first factor, respectively. Accordingly, (100-*a*)%, (100-*b*)% and (100-*c*)% of the three two-way interactions are allocated to the second factor.

In addition, (2) suppose d_1%, d_2%, and (100- d_1-d_2)% of the three-way interaction are allocated to population size, age structure, and DALY change, respectively.

Using A (A'), M (M') and P (P') to represent the number of DALYs attributed to age structure, DALY change, and population size defined by the method when using the year 1990 (year 2019) as a reference, the contributions of the three factors can be calculated as follows:

$$P = M_p + a\%I_{pa} + b\%I_{pm} + d_1\%I_{pam}$$

$$A = M_a + (100 - a)\%I_{pa} + \mathrm{c}\%I_{am} + d_2\%I_{pam}$$

$$M = M_m + (100 - b)\%I_{pm} + (100 - \mathrm{c})\%I_{am} + (100 - d_1 - d_2)\%I_{pam}$$

$$P' = M'_p + a\%I'_{pa} + b\%I'_{pm} + d_1\%I'_{pam}$$

$$A' = M'_a + (100 - a)\%I'_{pa} + c\%I'_{am} + d_2\%I'_{pam}$$

$$M' = M'_m + (100 - b)\%I'_{pm} + (100 - c)\%I'_{sm} + (100 - d_1 - d_2)\%I'_{pam}$$

The decomposition results should remain unchanged in absolute value when the reference population changes, so we have a group of three equations:

$$\begin{cases} P \equiv -P' \\ A \equiv -A' \\ M \equiv -M' \end{cases}$$

Through formula derivation, we have three simplified equations:

$$\begin{cases} \sum_{i=1}^{p}(N_2 - N_1)[(s_{i1}m_{i1} - s_{i2}m_{i2})(100 - a - b)\% + (s_{i2}m_{i1} - s_{i1}m_{i2})(a - b)\%] \equiv 0 \\ \sum_{i=1}^{p}(s_{i2} - s_{i1})[(N_2m_{i1} - N_1m_{i2})(100 - a - c)\% + (N_1m_{i1} - N_2m_{i2})(a - c)\%] \equiv 0 \\ \sum_{i=1}^{p}(m_{i2} - m_{i1})[(N_2s_{i2} - N_1s_{i1})(100 - b - c)\% + (N_1s_{i2} - N_2s_{i1})(b - c)\%] \equiv 0 \end{cases}$$

These three equations cannot always be true unless *a*, *b*, and *c* all equal 50.

The three equations have no requirements for d_1 and d_2. Given that there is no theoretical guidance for allocating the three-way interaction of three factors, we divide it equally, d_1=d_2=⅓×100.

The contributions of the three factors can be calculated as follows:

$$A = M_a + ½I_{am} + ½I_{pa} + ⅓I_{pam}$$

$$P = M_p + ½I_{pm} + ½I_{pa} + ⅓I_{pam}$$

$$M = M_m + ½I_{pm} + ½I_{am} + ⅓I_{pam}$$

Thus, *A* represents the effect of changes in age structure. Because the proportion of older age groups has been reported to increase recently for most countries [2], the impact of age structure represents the effect of population aging [3-6].

References

1. Cheng XJ, Tan LH, Gao YY, et al. A new method to attribute differences in total deaths between groups to population size, age structure and age-specific mortality rate. PLoS One 2019;14:e0216613. doi: 10.1371/journal.pone.0216613.

2. Lutz W, Sanderson W, Scherbov S. The coming acceleration of global population ageing. Nature 2008;451:716–19. doi: 10.1038/nature06516.

3. Chang AY, Skirbekk VF, Tyrovolas S, Kassebaum NJ, Dieleman JL. Measuring population ageing: an analysis of the Global Burden of Disease Study 2017. Lancet Public Health 2019;4:e159–e67. doi: 10.1016/S2468-2667(19)30019-2.

4. Cheng X, Yang Y, Schwebel DC, et al. Population ageing and mortality during 1990-2017: A global decomposition analysis. PLoS Med 2020;17:e1003138. doi: 10.1371/journal.pmed.1003138.

5. Zhang J, Pan L, Guo Q, et al. The impact of global, regional, and national population ageing on disability-adjusted life years and deaths associated with diabetes during 1990-2019: A global decomposition analysis. Diabetes Metab Syndr 2023;17:102791. doi: 10.1016/j.dsx.2023.102791.

6. Global Burden of Disease 2019 Cancer Collaboration; Kocarnik JM, Compton K, et al. Cancer Incidence, Mortality, Years of Life Lost, Years Lived With Disability, and Disability-Adjusted Life Years for 29 Cancer Groups From 2010 to 2019: A Systematic Analysis for the Global Burden of Disease Study 2019. JAMA Oncol 2022;8:420–44. doi:10.1001/jamaoncol.2021.6987.

Table S2 Checklist of information that should be included in new reports of global health estimates

Item #	Checklist item	Reported on page #
**Objectives and funding**		
**1**	Define the indicator(s), populations (including age, sex, and geographic entities), and time period(s) for which estimates were made.	Methods-paragraph 1 and 2
**2**	List the funding sources for the work.	No funding
**Data Inputs**		
For all data inputs from multiple sources that are synthesized as part of the study:		
**3**	Describe how the data were identified and how the data were accessed.	Methods-paragraph 1
**4**	Specify the inclusion and exclusion criteria. Identify all ad-hoc exclusions.	No data was excluded (Methods-paragraph 2)
**5**	Provide information on all included data sources and their main characteristics. For each data source used, report reference information or contact name/institution, population represented, data collection method, year(s) of data collection, sex and age range, diagnostic criteria or measurement method, and sample size, as relevant.	Methods-paragraph 1 and 2. Results-paragraph 1
**6**	Identify and describe any categories of input data that have potentially important biases (e.g., based on characteristics listed in item 5).	No such data (Methods-paragraph 1)
For data inputs that contribute to the analysis but were not synthesized as part of the study:		
**7**	Describe and give sources for any other data inputs.	All data were derived from GBD 2019 (Methods-paragraph 1 and 2)
For all data inputs:		
**8**	Provide all data inputs in a file format from which data can be efficiently extracted (e.g., a spreadsheet rather than a PDF), including all relevant meta-data listed in item 5. For any data inputs that cannot be shared because of ethical or legal reasons, such as third-party ownership, provide a contact name or the name of the institution that retains the right to the data.	Available from: http://ghdx.healthdata.org/gbd-results-tool and https://population.un.org/wpp/Download/Standard/Population/
**Data analysis**		
**9**	Provide a conceptual overview of the data analysis method. A diagram may be helpful.	Methods-paragraph 4 and S1 Text
**10**	Provide a detailed description of all steps of the analysis, including mathematical formulae. This description should cover, as relevant, data cleaning, data pre-processing, data adjustments and weighting of data sources, and mathematical or statistical	Methods-paragraph 4,5 6 and 7

	model(s).	
**11**	Describe how candidate models were evaluated and how the final model(s) were selected.	Only one method was used in this study (Methods-paragraph 4)
**12**	Provide the results of an evaluation of model performance, if done, as well as the results of any relevant sensitivity analysis.	The robust of the method was evaluated in a other study (S1 Text)
**13**	Describe methods for calculating uncertainty of the estimates. State which sources of uncertainty were, and were not, accounted for in the uncertainty analysis.	Discussion-limitations
**14**	State how analytic or statistical source code used to generate estimates can be accessed.	No specific software was needed for the estimation.
**Results and Discussion**		
**15**	Provide published estimates in a file format from which data can be efficiently extracted.	Results (Table 1-3, Figure 1-4) and supplementary (Table S1-S11, Figure S1-S4)
**16**	Report a quantitative measure of the uncertainty of the estimates (e.g. uncertainty intervals).	Discussion-limitations
**17**	Interpret results in light of existing evidence. If updating a previous set of estimates, describe the reasons for changes in estimates.	Discussion
**18**	Discuss limitations of the estimates. Include a discussion of any modelling assumptions or data limitations that affect interpretation of the estimates.	Discussion-limitations

This checklist should be used in conjunction with the GATHER statement and Explanation and Elaboration document, found on gather-statement.org

Table S3 The number (100000) and proportion of cancer-related DALYs attributed to population aging globally and by SDI from 1991 to 2019.

Year	Global	High SDI	High-middle SDI	Middle SDI	Low-middle SDI	Low SDI
1991	553656.44 (0.37)	233799.30 (0.59)	234040.26 (0.51)	264763.75 (0.65)	32993.83 (0.18)	-25819.31 (-0.36)
1992	1176683.99 (0.78)	506103.62 (1.27)	484882.47 (1.05)	561780.39 (1.39)	67138.03 (0.37)	-61010.90 (-0.85)
1993	1805639.06 (1.19)	794591.52 (2.00)	697513.41 (1.51)	880743.49 (2.17)	102764.31 (0.57)	-99747.92 (-1.40)
1994	2468812.06 (1.63)	1071422.85 (2.69)	908343.31 (1.97)	1236677.07 (3.05)	143825.38 (0.80)	-138299.77 (-1.94)
1995	3092087.93 (2.04)	1349792.09 (3.39)	1099852.64 (2.39)	1592030.33 (3.93)	178599.09 (0.99)	-180845.21 (-2.53)
1996	3802657.53 (2.51)	1607092.50 (4.04)	1335717.49 (2.90)	1981938.70 (4.89)	231214.35 (1.28)	-217798.69 (-3.05)
1997	4570524.75 (3.01)	1926701.57 (4.84)	1582836.81 (3.43)	2360134.19 (5.83)	285222.73 (1.58)	-254678.66 (-3.57)
1998	5351643.00 (3.53)	2273941.80 (5.71)	1805043.88 (3.92)	2753219.83 (6.80)	335263.18 (1.85)	-288505.47 (-4.04)
1999	6196450.02 (4.09)	2623496.55 (6.59)	2044049.48 (4.43)	3179449.04 (7.85)	390408.19 (2.16)	-317984.50 (-4.45)
2000	6973592.45 (4.60)	2967280.63 (7.46)	2251219.61 (4.88)	3584841.52 (8.85)	448217.74 (2.48)	-349630.50 (-4.90)
2001	7825543.58 (5.16)	3294922.15 (8.28)	2493429.53 (5.41)	3996501.56 (9.87)	538842.96 (2.98)	-375109.00 (-5.25)
2002	8714962.68 (5.75)	3685517.67 (9.26)	2728270.91 (5.92)	4398430.39 (10.86)	639355.22 (3.54)	-399592.92 (-5.60)
2003	9645300.72 (6.36)	4081005.62 (10.25)	2959360.73 (6.42)	4830652.25 (11.93)	746349.29 (4.13)	-419531.54 (-5.88)
2004	10715374.56 (7.06)	4448232.94 (11.18)	3249732.41 (7.05)	5340350.50 (13.19)	870516.97 (4.82)	-433739.33 (-6.08)
2005	11891813.86 (7.84)	4819336.87 (12.11)	3594055.10 (7.80)	5866697.76 (14.49)	1013479.46 (5.61)	-446077.41 (-6.25)
2006	13215651.32 (8.71)	5143011.42 (12.92)	4002230.89 (8.68)	6450839.40 (15.93)	1181340.71 (6.54)	-446388.06 (-6.25)
2007	14736258.40 (9.72)	5519772.75 (13.87)	4513291.28 (9.79)	7091533.59 (17.51)	1356889.89 (7.51)	-447405.54 (-6.27)
2008	16424328.07 (10.83)	5901118.57 (14.83)	5082588.19 (11.03)	7830097.14 (19.33)	1544028.17 (8.54)	-446111.47 (-6.25)

2009	18245554.08 (12.03)	6288424.30 (15.80)	5687380.70 (12.34)	8657484.80 (21.38)	1729858.27 (9.57)	-445454.01 (-6.24)
2010	20166521.49 (13.30)	6710473.62 (16.86)	6357480.00 (13.79)	9511230.08 (23.48)	1927635.13 (10.66)	-444582.58 (-6.23)
2011	22210782.03 (14.64)	7131308.29 (17.92)	7065435.82 (15.33)	10399175.17 (25.68)	2147890.57 (11.88)	-434798.31 (-6.09)
2012	24370412.47 (16.07)	7607689.92 (19.12)	7817801.99 (16.96)	11314192.29 (27.94)	2373797.37 (13.13)	-426208.50 (-5.97)
2013	26576236.04 (17.52)	8098985.11 (20.35)	8577585.05 (18.61)	12247923.87 (30.24)	2613535.17 (14.46)	-416764.15 (-5.84)
2014	28876173.79 (19.04)	8576308.27 (21.55)	9410128.42 (20.42)	13225383.60 (32.66)	2855717.67 (15.80)	-404534.21 (-5.67)
2015	31273964.37 (20.62)	9069038.38 (22.79)	10308827.98 (22.37)	14216233.33 (35.10)	3117656.28 (17.25)	-391449.26 (-5.48)
2016	33700951.32 (22.22)	9539492.45 (23.97)	11198474.03 (24.30)	15245353.53 (37.64)	3399149.01 (18.80)	-373468.70 (-5.23)
2017	36130512.80 (23.82)	10052012.53 (25.26)	12082314.88 (26.21)	16252731.37 (40.13)	3690953.42 (20.42)	-357697.82 (-5.01)
2018	38699388.66 (25.52)	10630491.86 (26.71)	13029131.37 (28.27)	17310957.62 (42.74)	3986011.59 (22.05)	-343074.77 (-4.81)
**2019**	**41377694.70 (27.28)**	**11212397.56 (28.17)**	**14025700.27 (30.43)**	**18451310.46 (45.56)**	**4287585.29 (23.72)**	**-326791.98 (-4.58)**

Table S4 The number (100000) and proportion of cancer-related DALYs attributed to population aging in males globally and by SDI from 1991 to 2019.

Year	Global	High SDI	High-middle SDI	Middle SDI	Low-middle SDI	Low SDI
1991	387270.69 (0.45)	165844.24 (0.74)	185285.73 (0.67)	155331.73 (0.67)	15399.93 (0.16)	-15160.12 (-0.44)
1992	816479.54 (0.95)	356525.89 (1.60)	381758.62 (1.39)	328421.13 (1.42)	31675.32 (0.34)	-35720.91 (-1.03)
1993	1249805.63 (1.46)	560535.08 (2.51)	551959.95 (2.01)	513996.44 (2.23)	48449.83 (0.52)	-58150.83 (-1.68)
1994	1704103.17 (1.99)	760510.29 (3.41)	721270.57 (2.63)	721121.18 (3.12)	66631.19 (0.71)	-80341.96 (-2.32)
1995	2128294.03 (2.48)	965734.21 (4.33)	873167.94 (3.18)	926606.85 (4.02)	79413.00 (0.85)	-105360.27 (-3.04)
1996	2607014.18 (3.04)	1159209.38 (5.20)	1057783.85 (3.85)	1155264.38 (5.01)	99984.78 (1.07)	-128396.75 (-3.7)
1997	3119735.85 (3.64)	1393317.7 (6.25)	1250786.94 (4.55)	1375825.98 (5.96)	118310.48 (1.26)	-151702.20 (-4.38)
1998	3635066.08 (4.24)	1649326.37 (7.39)	1422274.73 (5.18)	1603173.58 (6.95)	132482.34 (1.41)	-173261.56 (-5.00)
1999	4200703.45 (4.90)	1907277.12 (8.55)	1611042.01 (5.86)	1858498.59 (8.05)	150289.65 (1.60)	-192189.44 (-5.54)
2000	4722722.49 (5.51)	2160674.84 (9.68)	1771992.84 (6.45)	2102236.02 (9.11)	169760.93 (1.81)	-213433.15 (-6.16)
2001	5293973.11 (6.17)	2404475.42 (10.78)	1957084.50 (7.12)	2355108.94 (10.21)	206627.23 (2.21)	-231896.90 (-6.69)
2002	5902727.63 (6.88)	2695059.79 (12.08)	2141795.82 (7.8)	2606269.70 (11.29)	248545.65 (2.65)	-249876.22 (-7.21)
2003	6532944.44 (7.62)	2990417.84 (13.40)	2314730.54 (8.43)	2873927.88 (12.45)	295954.36 (3.16)	-265850.97 (-7.67)
2004	7244589.94 (8.45)	3264369.22 (14.63)	2523561.51 (9.19)	3191301.27 (13.83)	353610.37 (3.77)	-278560.13 (-8.03)
2005	8004580.31 (9.34)	3545824.06 (15.89)	2756888.83 (10.03)	3509227.13 (15.21)	418590.33 (4.47)	-290756.40 (-8.39)
2006	8840134.40 (10.31)	3781934.58 (16.95)	3029819.64 (11.03)	3855192.79 (16.71)	498933.46 (5.32)	-295289.11 (-8.52)
2007	9805419.74 (11.44)	4048823.13 (18.15)	3377140.74 (12.29)	4240687.87 (18.38)	584784.40 (6.24)	-301439.25 (-8.69)
2008	10871404.60 (12.68)	4309418.71 (19.32)	3763186.55 (13.70)	4689138.66 (20.32)	676975.72 (7.22)	-305352.47 (-8.81)

2009	12020904.74 (14.02)	4575472.88 (20.51)	4172537.01 (15.19)	5196095.38 (22.52)	767046.95 (8.19)	-310588.67 (-8.96)
2010	13223757.31 (15.42)	4880614.44 (21.88)	4621708.79 (16.82)	5709039.03 (24.74)	858759.35 (9.16)	-316185.17 (-9.12)
2011	14488479.11 (16.90)	5174369.88 (23.19)	5090722.41 (18.53)	6237683.86 (27.03)	965436.11 (10.30)	-314714.70 (-9.08)
2012	15838746.00 (18.47)	5508043.13 (24.69)	5592091.31 (20.35)	6790746.26 (29.43)	1076797.79 (11.49)	-313011.92 (-9.03)
2013	17186450.70 (20.05)	5851245.67 (26.23)	6087435.39 (22.16)	7331716.91 (31.77)	1193259.26 (12.73)	-311839.00 (-8.99)
2014	18580562.66 (21.67)	6181665.52 (27.71)	6636218.02 (24.15)	7892821.5 (34.20)	1305750.50 (13.94)	-307701.35 (-8.88)
2015	20057328.78 (23.39)	6534187.23 (29.29)	7237492.09 (26.34)	8467049.57 (36.69)	1425289.18 (15.21)	-304466.65 (-8.78)
2016	21558197.38 (25.15)	6863315.19 (30.76)	7831381.55 (28.51)	9062345.80 (39.27)	1566375.17 (16.72)	-297123.56 (-8.57)
2017	23055223.50 (26.89)	7229288.61 (32.40)	8410933.66 (30.61)	9630844.88 (41.74)	1716271.29 (18.32)	-291894.72 (-8.42)
2018	24643154.14 (28.74)	7640184.48 (34.24)	9029915.18 (32.87)	10234151.88 (44.35)	1866909.41 (19.92)	-288128.02 (-8.31)
**2019**	**26313152.17 (30.69)**	**8038183.89 (36.03)**	**9699431.95 (35.30)**	**10903786.44 (47.25)**	**2019497.03 (21.55)**	**-283696.80 (-8.18)**

Table S5 The number (100000) and proportion of cancer-related DALYs attributed to population aging in females globally and by SDI from 1991 to 2019.

Year	Global	High SDI	High-middle SDI	Middle SDI	Low-middle SDI	Low SDI
1991	212748.97 (0.32)	90115.18 (0.52)	79722.65 (0.43)	115226.73 (0.66)	16286.90 (0.19)	-11010.21 (-0.30)
1992	453047.18 (0.69)	195619.38 (1.12)	163883.93 (0.88)	244641.23 (1.40)	33048.20 (0.38)	-26124.86 (-0.71)
1993	695121.13 (1.05)	305748.64 (1.75)	235536.52 (1.27)	382796.50 (2.20)	50380.64 (0.58)	-43256.58 (-1.18)
1994	952316.83 (1.44)	409615.96 (2.34)	308455.82 (1.66)	537408.10 (3.08)	71119.38 (0.82)	-60753.28 (-1.65)
1995	1197894.69 (1.82)	512159.36 (2.93)	376843.74 (2.02)	691778.90 (3.97)	90232.10 (1.04)	-79640.05 (-2.17)
1996	1473982.39 (2.24)	603893.55 (3.45)	456952.97 (2.45)	857701.38 (4.92)	118974.71 (1.37)	-95108.78 (-2.59)
1997	1772795.25 (2.69)	717858.53 (4.11)	539529.37 (2.90)	1020733.34 (5.86)	151073.30 (1.74)	-110462.28 (-3.01)
1998	2082632.54 (3.16)	839502.66 (4.80)	617669.09 (3.32)	1191811.81 (6.84)	183569.10 (2.11)	-124534.12 (-3.39)
1999	2410271.15 (3.66)	962020.39 (5.50)	699222.88 (3.76)	1369181.40 (7.86)	218291.79 (2.51)	-136808.86 (-3.73)
2000	2713256.90 (4.11)	1084802.57 (6.20)	770957.32 (4.14)	1536652.96 (8.82)	254208.44 (2.92)	-148994.26 (-4.06)
2001	3039526.47 (4.61)	1200389.95 (6.87)	852642.42 (4.58)	1700640.66 (9.76)	305283.22 (3.51)	-157641.08 (-4.29)
2002	3365176.22 (5.10)	1333209.79 (7.62)	923993.89 (4.96)	1856815.22 (10.66)	362099.85 (4.16)	-165518.45 (-4.51)
2003	3711027.31 (5.63)	1466326.32 (8.39)	1000335.95 (5.37)	2026022.79 (11.63)	419952.36 (4.82)	-170952.02 (-4.66)
2004	4119584.81 (6.25)	1591164.83 (9.10)	1103156.57 (5.93)	2225488.81 (12.77)	485140.50 (5.57)	-173828.57 (-4.74)
2005	4581475.90 (6.95)	1716820.39 (9.82)	1231002.57 (6.61)	2437648.70 (13.99)	561618.94 (6.45)	-175159.65 (-4.77)
2006	5105263.37 (7.74)	1833047.80 (10.48)	1380755.34 (7.42)	2676534.35 (15.36)	647182.64 (7.43)	-171795.76 (-4.68)
2007	5701922.30 (8.65)	1970419.46 (11.27)	1566192.58 (8.41)	2933201.49 (16.83)	735315.00 (8.45)	-167780.93 (-4.57)

2008	6366039.33 (9.65)	2115544.88 (12.10)	1770517.79 (9.51)	3225442.02 (18.51)	827687.22 (9.51)	-163317.32 (-4.45)
2009	7081880.52 (10.74)	2260936.35 (12.93)	1988658.26 (10.68)	3549751.41 (20.37)	920591.15 (10.57)	-158421.34 (-4.32)
2010	7837177.51 (11.89)	2407587.22 (13.77)	2228691.38 (11.97)	3890141.15 (22.33)	1023914.22 (11.76)	-152878.02 (-4.16)
2011	8646495.34 (13.11)	2560575.31 (14.64)	2486901.33 (13.36)	4245922.15 (24.37)	1135339.63 (13.04)	-145054.51 (-3.95)
2012	9489744.64 (14.39)	2732388.44 (15.63)	2759101.37 (14.82)	4603951.86 (26.42)	1248273.16 (14.34)	-137859.17 (-3.76)
2013	10380275.06 (15.74)	2909843.75 (16.64)	3039888.21 (16.33)	4992306.21 (28.65)	1370979.70 (15.75)	-129564.81 (-3.53)
2014	11322974.53 (17.17)	3083839.76 (17.64)	3346847.12 (17.98)	5406435.20 (31.03)	1500251.06 (17.23)	-120628.08 (-3.29)
2015	12276926.57 (18.62)	3254774.11 (18.61)	3668591.07 (19.7)	5817263.94 (33.39)	1641703.39 (18.86)	-110277.62 (-3.00)
2016	13238479.23 (20.08)	3422313.38 (19.57)	3990459.21 (21.43)	6245290.54 (35.84)	1780439.27 (20.45)	-98857.71 (-2.69)
2017	14207326.30 (21.55)	3599046.78 (20.58)	4319271.26 (23.20)	6677780.16 (38.33)	1920876.38 (22.06)	-87593.92 (-2.39)
2018	15225736.90 (23.09)	3800560.50 (21.74)	4672597.84 (25.10)	7125769.23 (40.90)	2063328.59 (23.70)	-75940.00 (-2.07)
**2019**	**16273749.94 (24.68)**	**4013070.83 (22.95)**	**5031468.25 (27.02)**	**7591500.02 (43.57)**	**2210025.51 (25.38)**	**-63247.56 (-1.72)**

Table S6 The number (100000) and proportion of cancer-related DALYs attributed to population aging in 204 countries and territories, 1990 and 2019.

204 countries and territories	Total	Males	Females
	Population aging (attributed proportion)	Population aging (attributed proportion)	Population aging (attributed proportion)
Afghanistan	-198802.08 (-61.62)	-127009.87 (-88.72)	-78455.81 (-43.72)
Albania	46064.43 (65.33)	33204.33 (73.69)	14227.85 (55.92)
Algeria	113702.36 (38.17)	60147.75 (38.67)	54880.42 (38.55)
American Samoa	454.67 (52.19)	242.93 (49.42)	213.79 (56.32)
Andorra	1352.21 (53.74)	1003.05 (56.89)	391.11 (51.94)
Angola	2533.56 (1.78)	-2062.34 (-2.77)	3953.40 (5.80)
Antigua and Barbuda	323.87 (19.30)	205.12 (21.33)	165.70 (23.12)
Argentina	109310.63 (7.81)	64533.21 (8.34)	48256.22 (7.72)
Armenia	39530.38 (32.33)	24217.72 (35.76)	16072.98 (29.46)
Australia	234731.64 (31.86)	165171.00 (39.72)	85103.92 (26.51)
Austria	81584.85 (18.01)	71784.37 (29.95)	23853.80 (11.19)
Azerbaijan	50910.32 (24.36)	33523.62 (27.28)	19061.65 (22.13)
Bahamas	3743.69 (50.67)	2233.42 (58.67)	1597.69 (44.60)
Bahrain	6864.48 (93.78)	4329.34 (101.26)	2591.74 (85.14)
Bangladesh	595289.46 (38.57)	337919.53 (37.13)	265048.55 (41.84)
Barbados	3324.37 (33.17)	1990.96 (39.86)	1491.46 (29.66)
Belarus	86632.53 (16.05)	66964.35 (21.04)	29446.42 (13.30)
Belgium	114698.47 (17.69)	96292.65 (25.5)	35808.07 (13.23)
Belize	516.07 (23.15)	265.94 (24.78)	295.96 (25.61)
Benin	-8998.46 (-16.84)	-8505.27 (-32.4)	-1799.40 (-6.62)
Bermuda	1608.31 (54.48)	1077.80 (64.87)	581.29 (45.04)
Bhutan	2545.42 (39.35)	1494.30 (47.87)	1078.41 (32.22)
Bolivia (Plurinational State of)	45681.61 (31.33)	20509.40 (34.13)	25371.63 (29.61)
Bosnia and Herzegovina	83090.67 (51.44)	57818.6 (60.01)	29333.63 (45.00)

Botswana	4393.04 (20.79)	686.54 (6.17)	3250.69 (32.5)
Brazil	1560438.01 (48.51)	826742.78 (48.62)	719957.66 (47.48)
Brunei Darussalam	3487.39 (53.28)	1666.67 (49.37)	1860.09 (58.69)
Bulgaria	79416.72 (17.24)	42766.83 (15.8)	34167.21 (17.97)
Burkina Faso	-25795.91 (-22.38)	-19345.66 (-39.05)	-7905.51 (-12.03)
Burundi	-5774.46 (-6.01)	667.14 (1.57)	-6598.47 (-12.27)
Cabo Verde	-517.49 (-8.95)	-861.89 (-32.18)	71.88 (2.32)
Cambodia	55643.32 (31.62)	20271.06 (24.38)	30853.82 (33.24)
Cameroon	-24454.22 (-18.56)	-17241.59 (-29.04)	-8208.53 (-11.34)
Canada	526210.13 (41.73)	346192.86 (50.01)	203651.16 (35.81)
Central African Republic	-2272.71 (-4.53)	-1685.11 (-6.72)	-1074.30 (-4.28)
Chad	-22444.59 (-33.52)	-9743.51 (-31.41)	-12710.02 (-35.36)
Chile	195570.37 (47.13)	102655.18 (49.99)	93757.77 (44.74)
China	24002880.61 (57.87)	15720137.26 (61.05)	8495821.66 (54.02)
Colombia	347985.65 (55.23)	152074.87 (51.17)	195839.29 (58.83)
Comoros	1438.61 (20.97)	316.08 (10.21)	1034.02 (27.47)
Congo	5818.07 (11.96)	3776.17 (16.17)	2144.43 (8.48)
Cook Islands	281.53 (57.39)	191.91 (64.53)	93.13 (48.21)
Costa Rica	32215.50 (54.44)	17022.54 (54.86)	15021.99 (53.36)
Coted'Ivoire	20273.24 (15.47)	8528.90 (11.57)	10914.86 (19.03)
Croatia	77610.59 (26.24)	58537.31 (32.58)	27217.23 (23.44)
Cuba	187277.08 (52.43)	101720.12 (52.2)	82262.26 (50.67)
Cyprus	6788.66 (31.02)	4401.11 (38.31)	2743.35 (26.38)
Czechia	165633.59 (24.11)	122266.62 (30.02)	57554.24 (20.57)
Democratic People's Republic of Korea	182272.30 (25.52)	110822.92 (28.44)	81987.95 (25.26)
Democratic Republic of the Congo	-40020.98 (-7.65)	-35370.50 (-14.46)	-16719.26 (-6.00)
Denmark	63657.29 (19.05)	46188.54 (27.22)	22373.51 (13.60)
Djibouti	3797.21 (72.25)	2331.32 (90.89)	1511.89 (56.19)

Dominica	480.62 (15.44)	484.26 (29.20)	118.04 (8.12)
Dominican Republic	44388.93 (50.10)	20754.98 (47.82)	22066.47 (48.82)
Ecuador	66682.71 (41.59)	28572.27 (39.34)	38390.99 (43.79)
Egypt	107418.98 (15.67)	91445.54 (22.27)	23641.02 (8.60)
El Salvador	22467.45 (32.09)	8412.97 (28.92)	14489.59 (35.40)
Equatorial Guinea	-4328.28 (-63.36)	-3443.90 (-100.62)	-1062.55 (-31.18)
Eritrea	3755.54 (9.30)	1696.16 (9.94)	1801.44 (7.72)
Estonia	18222.61 (20.26)	13325.95 (26.65)	7132.93 (17.86)
Eswatini	1997.06 (17.13)	36.05 (0.58)	1407.47 (25.63)
Ethiopia	-72061.19 (-9.46)	-46412.17 (-14.42)	-25007.47 (-5.69)
Fiji	5071.09 (38.21)	1976.07 (37.74)	3003.49 (37.38)
Finland	74204.33 (32.02)	58866.18 (47.63)	24544.61 (22.70)
France	839910.74 (25.78)	658644.41 (31.75)	265376.2 (22.42)
Gabon	-876.74 (-4.03)	507.61 (4.35)	-837.19 (-8.31)
Gambia	751.89 (8.28)	-111.78 (-2.01)	757.06 (21.56)
Georgia	38422.49 (16.29)	25303.41 (20.05)	15317.56 (13.97)
Germany	1096964.25 (24.14)	921227.18 (38.01)	347515.31 (16.39)
Ghana	17404.53 (8.24)	37.35 (0.04)	17137.93 (14.21)
Greece	151364.82 (28.80)	96504.01 (30.59)	58395.23 (27.81)
Greenland	1908.50 (66.08)	1328.75 (81.42)	653.21 (51.99)
Grenada	360.14 (11.94)	213.68 (13.58)	161.94 (11.24)
Guam	1736.66 (68.92)	1119.72 (69.78)	628.56 (68.67)
Guatemala	12785.06 (11.59)	2698.08 (5.86)	11668.5 (18.16)
Guinea	-29007.07 (-23.56)	-14901.38 (-25.42)	-13487.82 (-20.92)
Guinea-Bissau	-2024.36 (-12.22)	-1856.55 (-22.77)	-465.52 (-5.54)
Guyana	4969.11 (34.32)	2183.54 (33.66)	2759.35 (34.53)
Haiti	82.44 (0.05)	-3241.84 (-4.81)	5114.58 (5.37)
Honduras	12451.81 (20.95)	4760.04 (18.88)	8005.96 (23.40)

Hungary	135212.51 (17.43)	84799.61 (18.67)	55243.01 (17.17)
Iceland	2750.53 (28.24)	1746.08 (34.98)	1099.12 (23.15)
India	3059869.90 (26.92)	1443878.04 (24.70)	1584175.63 (28.69)
Indonesia	1031609.34 (35.92)	462692.36 (37.81)	560957.07 (34.03)
Iran (Islamic Republic of)	327379.46 (44.38)	167336.51 (39.00)	153050.20 (49.60)
Iraq	18225.40 (8.32)	4480.03 (3.83)	13628.40 (13.38)
Ireland	34701.00 (21.15)	24145.88 (27.72)	13149.18 (17.08)
Israel	34868.50 (21.93)	18003.47 (23.01)	16796.49 (20.81)
Italy	907461.94 (26.49)	638839.31 (31.13)	334424.36 (24.34)
Jamaica	11249.00 (24.01)	5909.91 (24.75)	5722.90 (24.91)
Japan	2873842.05 (50.86)	2054392.2 (60.52)	1002403.68 (44.43)
Jordan	22529.23 (54.30)	11694.16 (58.28)	10682.61 (49.87)
Kazakhstan	92411.33 (14.16)	71716.88 (19.27)	30682.30 (10.94)
Kenya	23883.93 (12.74)	3659.88 (4.25)	18912.68 (18.66)
Kiribati	302.57 (11.93)	104.15 (9.50)	198.19 (13.76)
Kuwait	8901.85 (59.02)	5345.14 (63.99)	4352.18 (64.67)
Kyrgyzstan	3741.63 (3.13)	4078.19 (6.07)	767.16 (1.47)
Lao People's Democratic Republic	3341.58 (3.78)	-305.23 (-0.71)	3380.78 (7.44)
Latvia	29188.48 (19.86)	20915.03 (25.56)	11324.44 (17.39)
Lebanon	19030.05 (24.25)	7402.48 (17.82)	10885.98 (29.47)
Lesotho	-1278.45 (-4.42)	-2944.44 (-18.15)	753.72 (5.94)
Liberia	-12695.87 (-43.84)	-9075.95 (-61.80)	-4001.99 (-28.04)
Libya	20210.99 (36.06)	10617.50 (32.24)	10072.42 (43.58)
Lithuania	48184.05 (26.19)	32347.50 (30.70)	18352.55 (23.35)
Luxembourg	1944.66 (8.22)	2394.53 (17.67)	276.82 (2.74)
Madagascar	-9013.68 (-5.37)	-11491.82 (-15.95)	1451.16 (1.52)
Malawi	-12741.48 (-8.82)	-8279.75 (-13.87)	-5117.72 (-6.04)
Malaysia	133752.86 (42.03)	68681.97 (43.44)	65666.99 (41)

Maldives	368.63 (13.72)	-282.94 (-21.75)	605.06 (43.69)
Mali	-32801.25 (-25.26)	-16595.92 (-26.76)	-16007.93 (-23.59)
Malta	6434.93 (44.88)	4288.79 (55.3)	2341.81 (35.58)
Marshall Islands	282.68 (36.02)	143.55 (33.93)	151.25 (41.83)
Mauritania	-244.70 (-0.80)	625.90 (4.61)	-583.03 (-3.41)
Mauritius	11757.33 (64.14)	6487.84 (69.68)	5323.56 (59.02)
Mexico	605564.21 (48.46)	262972.6 (47.82)	343892.94 (49.14)
Micronesia (Federated States of)	539.55 (24.09)	218.71 (18.73)	316.39 (29.53)
Monaco	288.86 (10.36)	263.12 (16.51)	70.41 (5.89)
Mongolia	26875.55 (34.37)	14068.01 (31.33)	11637.12 (34.94)
Montenegro	8811.63 (34.62)	6291.44 (40.99)	2913.53 (28.84)
Morocco	139078.58 (42.98)	67270.54 (42.34)	70651.92 (42.90)
Mozambique	-42065.08 (-27.31)	-25045.68 (-43.59)	-18133.37 (-18.78)
Myanmar	243882.37 (24.11)	103063.81 (23.95)	137157.86 (23.59)
Namibia	1129.59 (6.61)	-416.27 (-5.11)	1356.66 (15.19)
Nauru	1.51 (0.63)	-8.63 (-6.71)	9.48 (8.69)
Nepal	56944.45 (20.11)	30517.75 (20.84)	28584.17 (20.91)
Netherlands	291292.93 (36.12)	221805.85 (47.89)	97589.70 (28.44)
New Zealand	56515.51 (35.35)	36994.27 (44.64)	22851.07 (29.68)
Nicaragua	16056.47 (40.91)	6107.33 (35.71)	9881.04 (44.61)
Niger	-3138.39 (-4.60)	-3969.95 (-12.72)	1625.32 (4.40)
Nigeria	-193322.79 (-22.54)	-97872.80 (-22.89)	-33463.89 (-7.78)
Niue	10.84 (15.24)	7.16 (19.97)	3.99 (11.31)
North Macedonia	28647.31 (35.84)	18040.26 (38.86)	10664.06 (31.82)
Northern Mariana Islands	1185.26 (110.22)	653.48 (104.85)	531.54 (117.57)
Norway	19249.82 (8.84)	17569.67 (14.95)	5304.15 (5.29)
Oman	-2737.68 (-16.58)	-1929.03 (-19.12)	-438.55 (-6.83)
Pakistan	-172757.85 (-8.51)	-126512.59 (-11.80)	-52061.48 (-5.43)

Palau	309.64 (59.10)	135.67 (52.04)	174.94 (66.46)
Palestine	168.47 (0.58)	502.69 (3.25)	-382.93 (-2.80)
Panama	17390.72 (40.73)	8734.29 (40.71)	8511.60 (40.07)
Papua New Guinea	4027.71 (6.46)	1434.59 (5.06)	3203.37 (9.43)
Paraguay	14048.34 (26.26)	7226.26 (29.53)	6933.04 (23.88)
Peru	157835.22 (38.55)	71783.40 (36.92)	87860.56 (40.87)
Philippines	326295.75 (29.34)	148900.52 (25.79)	170123.34 (31.81)
Poland	596287.68 (27.95)	417419.38 (33.29)	215689.85 (24.53)
Portugal	153044.27 (30.47)	101164.47 (35.55)	59181.57 (27.19)
Puerto Rico	47996.94 (45.82)	27699.54 (46.27)	19596.47 (43.65)
Qatar	2063.55 (47.66)	1683.78 (63.92)	826.06 (48.73)
Republic of Korea	1106434.43 (77.44)	816186.15 (94.25)	360639.17 (64.10)
Republic of Moldova	34766.73 (18.93)	21771.66 (21.04)	13649.57 (17.02)
Romania	235184.85 (23.67)	146022.28 (25.46)	92879.52 (22.12)
Russian Federation	1288266.64 (17.18)	1073805.47 (25.08)	430860.95 (13.39)
Rwanda	7191.92 (5.39)	-327.15 (-0.59)	6386.97 (8.16)
Saint Kitts and Nevis	190.11 (10.80)	90.43 (11.37)	112.92 (11.71)
Saint Lucia	1821.79 (49.85)	1090.54 (58.64)	794.45 (44.26)
Saint Vincent and the Grenadines	1262.63 (45.76)	816.72 (63.06)	515.44 (35.20)
Samoa	534.00 (18.49)	272.76 (18.62)	242.79 (17.06)
San Marino	354.88 (27.31)	294.82 (36.07)	104.50 (21.69)
Sao Tome and Principe	-351.73 (-20.23)	-253.01 (-30.52)	-112.46 (-12.36)
Saudi Arabia	25958.81 (21.15)	11191.21 (15.96)	16646.14 (31.62)
Senegal	4109.81 (4.68)	-3029.14 (-7.03)	6357.57 (14.24)
Serbia	118265.41 (23.1)	71006.85 (23.91)	48744.73 (22.67)
Seychelles	722.02 (31.88)	432.54 (33.20)	309.93 (32.22)
Sierra Leone	-13280.76 (-29.23)	-9135.33 (-37.61)	-4829.03 (-22.84)
Singapore	63223.67 (69.30)	40652.89 (78.39)	23969.54 (60.88)

Slovakia	71613.81 (26.28)	48512.82 (28.37)	25454.95 (25.08)
Slovenia	36945.53 (36.40)	28387.73 (50.17)	12557.59 (27.96)
Solomon Islands	1135.99 (15.17)	131.88 (3.31)	999.09 (28.49)
Somalia	-19913.53 (-19.48)	-12931.87 (-28.82)	-8690.61 (-15.15)
South Africa	220889.26 (27.73)	106197.47 (26.06)	109859.40 (28.25)
South Sudan	5780.39 (7.76)	3073.19 (7.64)	4101.82 (11.98)
Spain	496035.02 (25.92)	359769.29 (30.03)	175148.72 (24.46)
Sri Lanka	126245.22 (53.42)	59076.77 (48.00)	64853.67 (57.26)
Sudan	-20523.00 (-9.36)	-13565.91 (-11.31)	-5553.63 (-5.59)
Suriname	3216.73 (41.32)	1642.51 (43.61)	1513.83 (37.67)
Sweden	48859.87 (11.00)	40303.19 (17.45)	14904.63 (6.98)
Switzerland	68553.18 (20.28)	56258.44 (28.95)	20952.75 (14.58)
Syrian Arab Republic	58480.93 (44.61)	35915.22 (50.35)	23579.45 (39.45)
Taiwan (Province of China)	406430.45 (69.45)	229212.49 (62.25)	164052.85 (75.59)
Tajikistan	1522.72 (1.43)	1931.30 (3.27)	622.34 (1.32)
Thailand	1030527.39 (71.51)	594333.94 (75.44)	438322.08 (67.10)
Timor-Leste	3904.06 (44.41)	2248.44 (56.14)	1608.46 (33.60)
Togo	10135.87 (26.63)	1930.79 (11.52)	7084.67 (33.25)
Tokelau	2.79 (6.49)	1.30 (7.84)	0.96 (3.63)
Tonga	424.14 (18.41)	230.46 (18.32)	173.97 (16.64)
Trinidad and Tobago	13939.38 (48.08)	7546.12 (53.15)	6412.87 (43.34)
Tunisia	57103.21 (54.02)	32303.23 (52.18)	23357.37 (53.34)
Turkey	589750.70 (39.22)	379734.28 (39.87)	206007.10 (37.37)
Turkmenistan	19985.73 (26.69)	10625.33 (26.41)	9501.22 (27.42)
Tuvalu	16.32 (5.63)	4.11 (3.26)	10.87 (6.65)
Uganda	-32749.89 (-15.62)	-33498.16 (-29.39)	-3639.84 (-3.80)
Ukraine	283155.70 (8.86)	224827.62 (12.37)	98448.03 (7.15)
United Arab Emirates	35113.51 (155.84)	24575.89 (162.25)	11040.46 (149.50)

United Kingdom	409991.13 (11.31)	359845.88 (18.93)	124809.02 (7.24)
United Republic of Tanzania	-5865.94 (-1.59)	-11325.64 (-6.34)	4486.54 (2.35)
United States of America	3268935.71 (26.15)	2236177.51 (33.57)	1219908.24 (20.89)
United States Virgin Islands	2640.44 (78.45)	1598.42 (93.12)	1033.20 (62.65)
Uruguay	18772.72 (9.74)	10470.99 (9.26)	8398.86 (10.54)
Uzbekistan	52303.59 (13.72)	30361.85 (14.63)	25020.17 (14.41)
Vanuatu	563.95 (24.92)	275.88 (20.92)	278.05 (29.45)
Venezuela (Bolivarian Republic of)	230231.54 (67.04)	124308.46 (77.40)	105977.57 (57.96)
Viet Nam	559494.20 (45.45)	303734.27 (44.98)	250729.78 (45.12)
Yemen	-4173.26 (-3.38)	-2931.51 (-4.27)	511.12 (0.93)
Zambia	-3732.60 (-2.98)	-10228.44 (-18.60)	3088.69 (4.38)
Zimbabwe	14175.23 (8.50)	-5957.56 (-7.04)	17818.60 (21.70)

Table S7 The number (100000) and proportion of cancer-related DALYs attributed to population aging in 30 cancer types, 1990-2019.

Cancer type	Total	Male	Female
	Population aging (attributed proportion)	Population aging (attributed proportion)	Population aging (attributed proportion)
Bladder cancer	950319.28 (37.10)	817917.81 (43.08)	215510.03 (32.51)
Brain and central nervous system cancer	650247.91 (16.40)	388341.23 (17.03)	267214.49 (15.85)
Breast cancer	3129526.32 (26.82)	56536.51 (36.65)	2953267.76 (25.65)
Cervical cancer	1248986.14 (20.23)	/	1222180.56 (19.79)
Colon and rectum cancer	4279594.29 (34.67)	2546709.73 (39.52)	1807777.09 (30.65)
Esophageal cancer	2422837.34 (29.53)	1872604.86 (32.65)	629618.74 (25.48)
Gallbladder and biliary tract cancer	730667.55 (33.97)	327389.47 (39.27)	396074.42 (30.07)
Hodgkin lymphoma	25442.94 (2.48)	26187.55 (4.06)	802.86 (0.21)
Kidney cancer	622009.82 (35.32)	441239.66 (39.95)	198478.06 (30.24)
Larynx cancer	667262.40 (26.99)	619775.77 (28.77)	79587.68 (24.99)
Leukemia	641134.30 (9.39)	413391.08 (10.98)	257974.61 (8.43)
Lip and oral cavity cancer	812401.06 (28.71)	597867.17 (29.65)	229335.51 (28.17)
Liver cancer	2382560.15 (21.94)	1760628.31 (22.41)	658865.50 (21.94)
Malignant skin melanoma	241043.98 (24.15)	147481.71 (27.04)	97816.23 (21.60)
Mesothelioma	116694.66 (29.89)	94706.60 (37.32)	29744.61 (21.76)
Multiple myeloma	452659.29 (37.03)	263905.64 (41.38)	194874.25 (33.34)
Nasopharynx cancer	329564.17 (18.08)	238829.29 (19.80)	94408.14 (15.30)
Non-Hodgkin lymphoma	780894.51 (22.50)	486579.21 (23.72)	309890.91 (21.84)
Non-melanoma skin cancer	212433.01 (38.03)	/	/
Other malignant neoplasms	966230.86 (14.69)	568254.62 (15.54)	417596.61 (14.29)
Other neoplasms	216651.19 (41.97)	143997.84 (48.68)	82424.95 (37.40)
Other pharynx cancer	476358.26 (30.62)	390365.72 (32.96)	96657.94 (26.01)

Ovarian cancer	802532.39 (29.71)	/	756360.57 (28.00)
Pancreatic cancer	1900167.47 (40.93)	1119182.94 (42.7)	793197.57 (39.24)
Prostate cancer	1946145.12 (44.75)	2337198.28 (53.74)	/
Stomach cancer	5054663.35 (24.72)	3559774.42 (27.84)	1650176.35 (21.53)
Testicular cancer	-20458.02 (-5.96)	-18334.92 (-5.34)	/
Thyroid cancer	169148.86 (26.05)	71691.29 (32.49)	94181.27 (21.97)
Tracheal, bronchus, and lung cancer	8723389.51 (32.20)	6894526.65 (33.88)	2251022.67 (33.36)
Uterine cancer	446586.57 (30.14)	/	413212.02 (27.88)

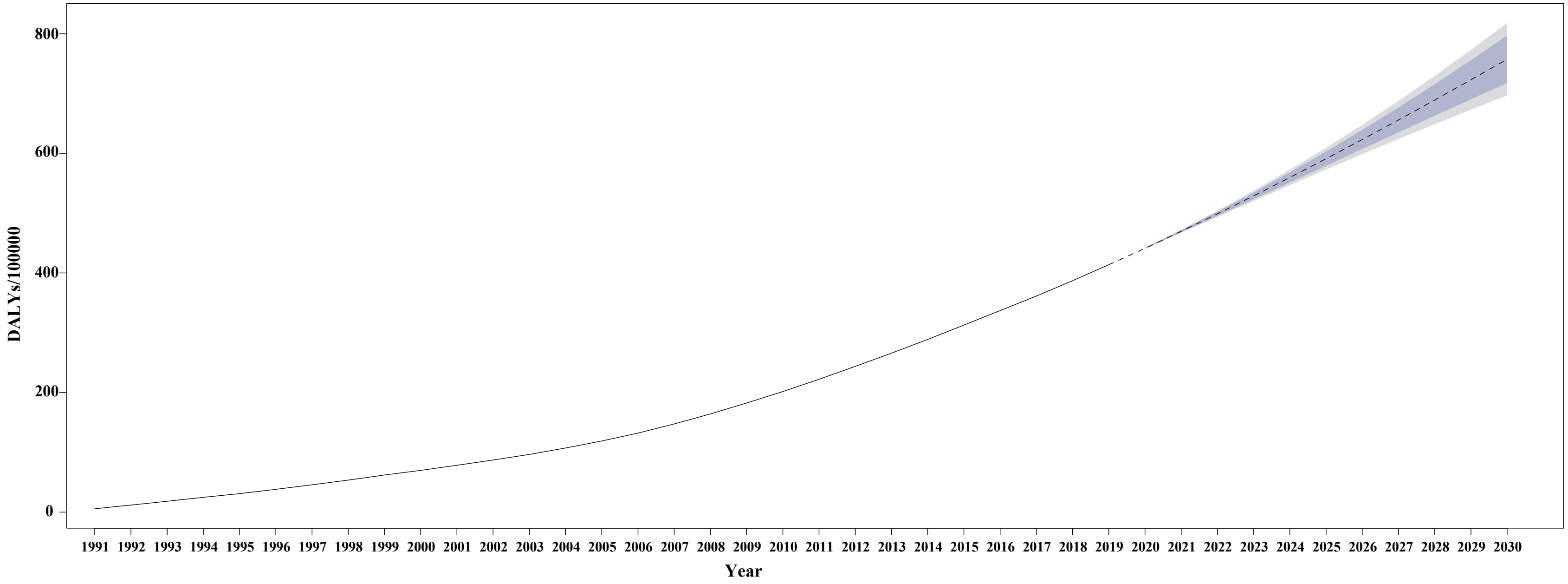

Figure S1 The forecast of the number (100000) cancer-related DALYs attributed to population aging globally by 2030.

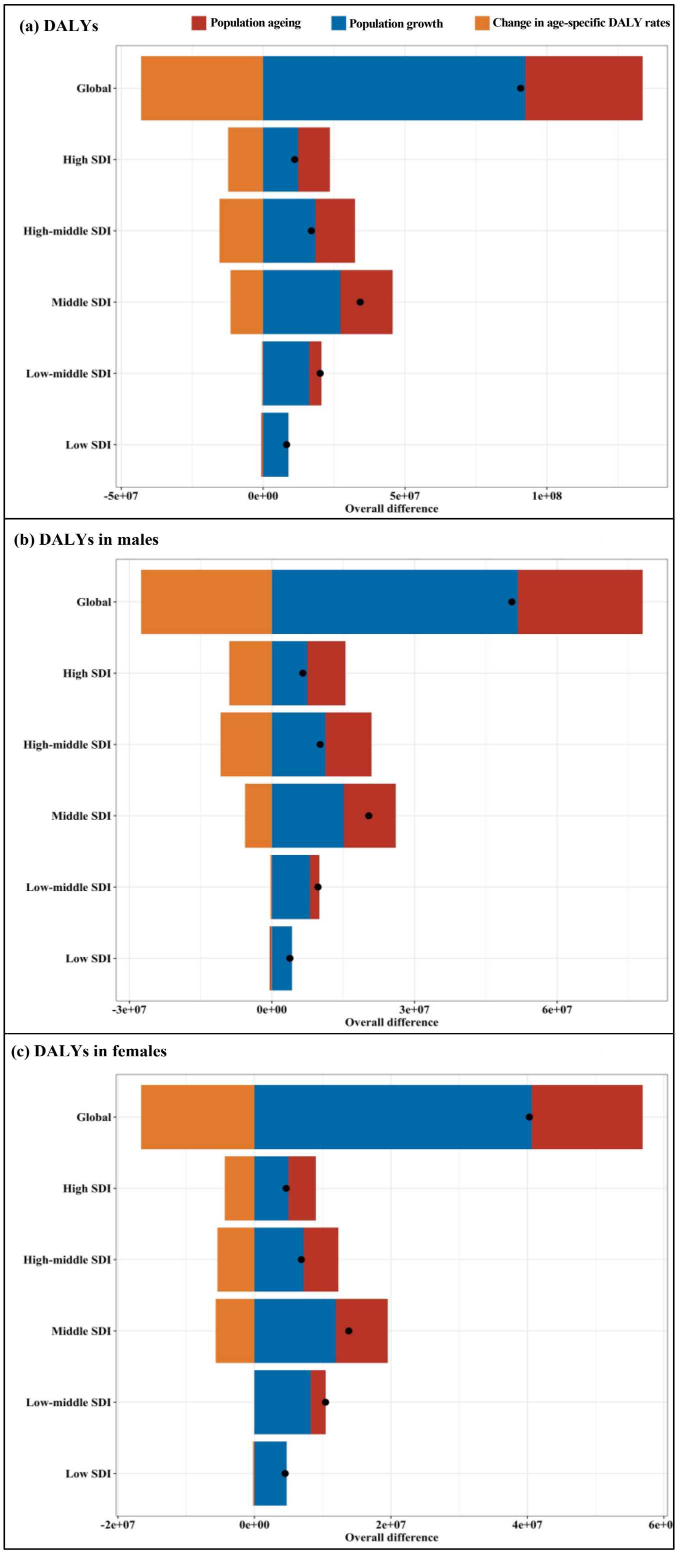

Figure S2 Cancer-related DALYs attributed to population aging, population growth, and change in age-specific DALY rates globally and by SDI, 1990-2019. DALYs (Panel a); DALYs in males (Panel b); DALYs in females (Panel c).

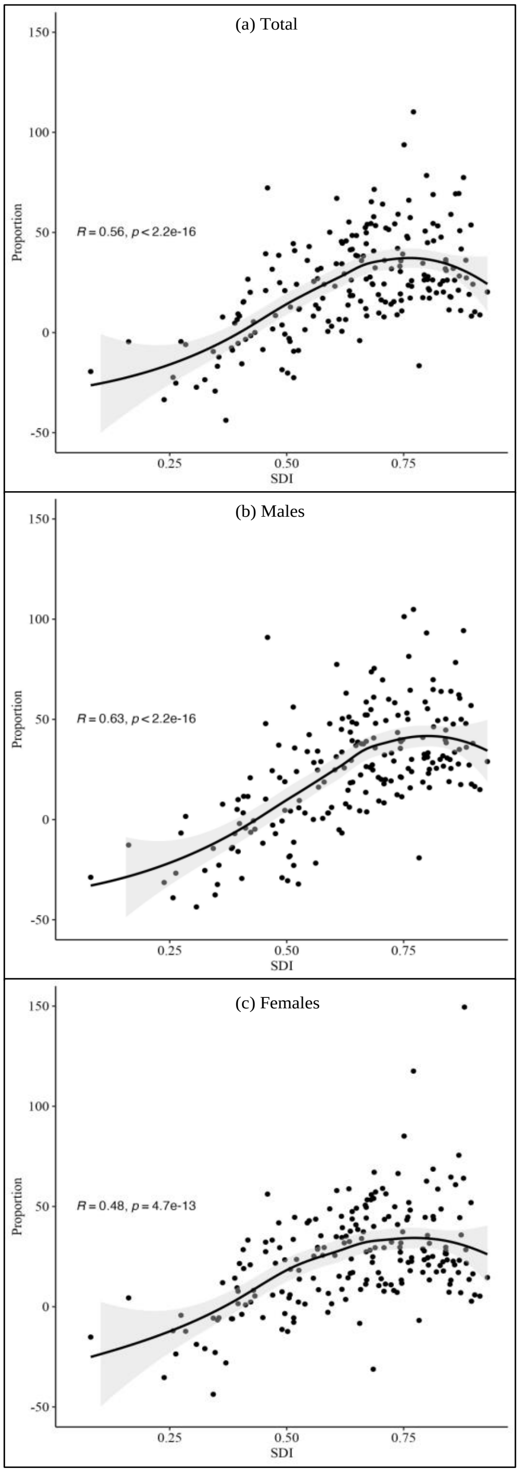

Figure S3 The association between cancer-related DALYs attributed to population aging in 204 countries and territories and SDI. Total (Panel a); Males (Panel b); Females (Panel c).

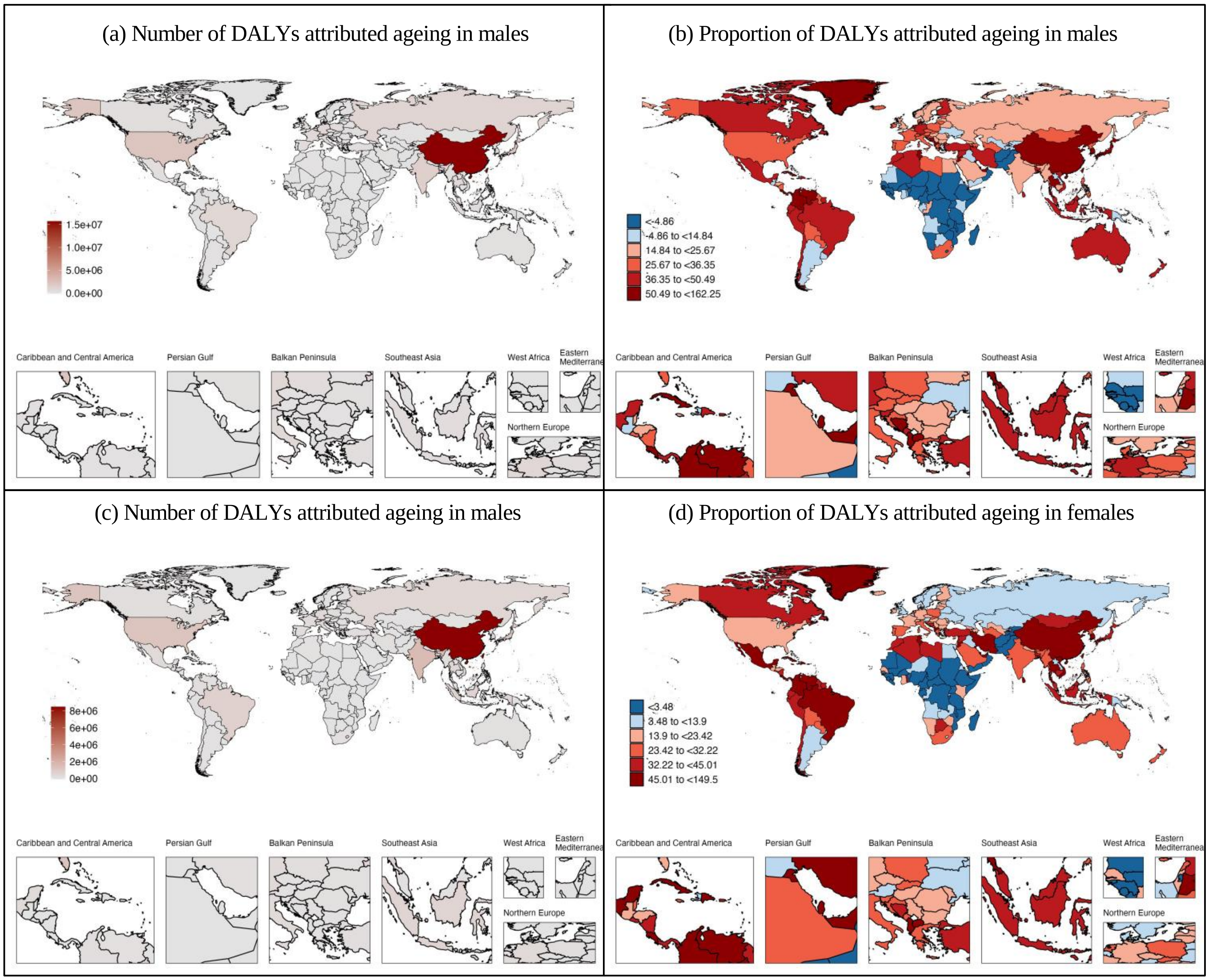

Figure S4 DALYs attributed to population ageing between 1990 and 2019 in 204 countries and territories. Number of DALYs attributed to aging in males (Panel a); Proportion of DALYs attributed to aging in males (Panel b); Number of DALYs attributed to aging in females (Panel c); Proportion of DALYs attributed to aging in females (Panel d).

Figure S5 Cancer-related DALYs attributed to population aging by SDI 1990-2019, stratified by 30 cancer types.

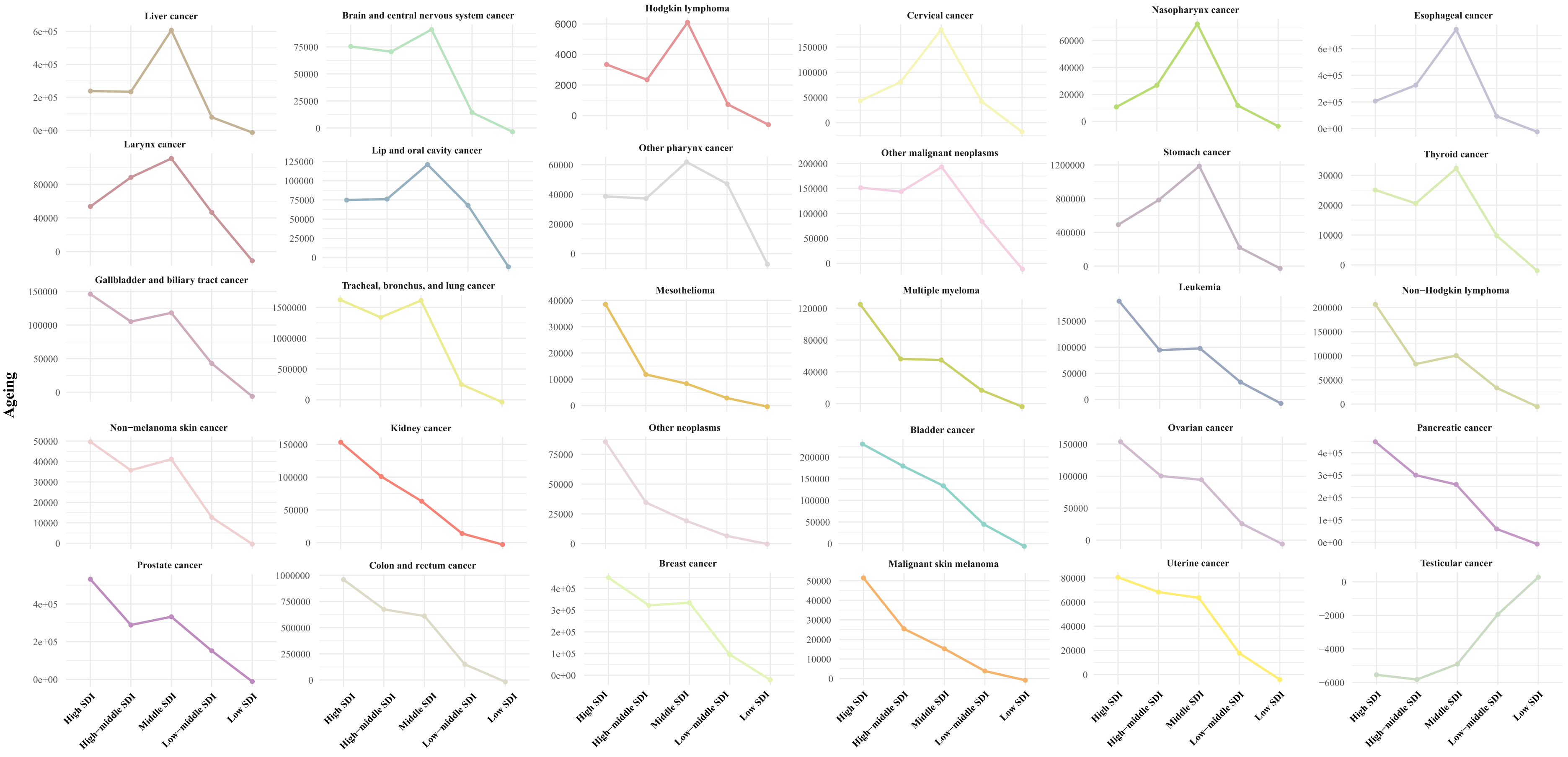

Supplement: Online Supplementary Document [file jogh-14-04144-s001.pdf]
